# Mitigating the Impact of Electrode Shift on Classification Performance in Electromyography Applications Using Sliding-Window Normalization

**DOI:** 10.3390/s25134119

**Published:** 2025-07-01

**Authors:** Taichi Tanaka, Isao Nambu, Yasuhiro Wada

**Affiliations:** 1 Department of Science Technology of Innovation, Nagaoka University of Technology, Nagaoka 940-2188, Japan; taichi_tanaka@stn.nagaokaut.ac.jp; 2 Department of Electrical, Electronics and Information Engineering, Nagaoka University of Technology, Nagaoka 940-2188, Japan; ywada@vos.nagaokaut.ac.jp

**Keywords:** electromyography, EMG, z-score, signal normalization, electrode shift, DNN classification

## Abstract

Electromyography (EMG) signals have diverse applications, ranging from prosthetic hands and assistive suits to rehabilitation devices. Nonetheless, their performance suffers from cross-subject generalization issues, electrode shifts, and daily variability. In a previous study, while transfer learning narrowed the classification performance gap to −1% in an eight-class scenario under electrode shift, they imposed the burden of additional data collection and re-training. To address this issue in real-time prediction, we investigated a sliding-window normalization (SWN) technique that merges z-score normalization with sliding-window processing to align the EMG amplitude across channels and mitigate the performance degradation caused by electrode displacement. We validated SWN using experimental data from a right-arm trajectory-tracking task involving three motion classes (rest, flexion, and extension of the elbow). Offline analysis revealed that SWN mitigated accuracy degradation to −1.0% without additional data for re-training or multi-condition training, a 6.6% improvement compared with the −7.6% baseline without normalization. The advantage of SWN is that it operates with data from a single electrode position for training, which eliminates both the collection of multi-position training data and the calibration of deep learning models before practical use in EMG applications. Moreover, combining SWN with multi-position training exceeded the classification accuracy of the no-shift condition by 2.4%.

## 1. Introduction

In the field of electromyography (EMG) for robot control and human motion prediction, researchers have investigated prosthetic hand control [1,2], gesture prediction [3,4], assist suit control [2], and rehabilitation [5]. A previous study used a simple threshold for control, whereas another study employed machine learning for control [2]. In machine learning approaches, time-domain features such as integrated EMG, waveform length, and root mean square [6] were extracted, whereas time-frequency features such as the spectrogram of short-time Fourier transform [7] and wavelets [8] were utilized. Compared with a simple threshold-based method, the advantage of machine learning is that it enables automatic classifier construction for the prediction of motion classes from handcrafted EMG features. However, handcrafted features pose challenges in determining the optimal window length for feature extraction and in selecting suitable feature combinations. Consequently, since 2016, most researchers have employed deep neural networks (DNNs) for human motion prediction [2,9]. DNNs automatically extract features from EMG signals, requiring only the window length to be set. Recent DNN models include convolutional long short-term memory (CNN-LSTM) [10] and transformer [3]. CNN-LSTM is a hybrid architecture that uses CNN layers to extract local spatiotemporal features from EMG signals and LSTM layers to capture the temporal evolution of these features. In contrast, the transformer is a deep learning architecture based on self-attention that enables the parallel processing of sequential data and has achieved state-of-the-art performance in tasks such as machine translation and language modeling.

Despite the high performance achieved by current machine learning and DNN methods, these approaches often require additional training data and calibration procedures, which impose considerable operational burden in real-world applications. Recent studies have focused on practical issues, such as cross-subject generalization [4,11,12], electrode shift [9,13,14,15,16], and daily effects [16,17,18], rather than solely improving machine learning performance [19,20]. These issues arise because EMG amplitude varies with differences in muscle mass, electrode placement, muscle fatigue, and skin impedance (skin thickness and temperature). To address these challenges, various methods have been proposed, including transfer learning (TL) [13,21], adversarial domain adaptation (ADA) [16,22], and cross-domain autoencoders for subject or session adaptation [4,11] and re-calibration [16,17,18]. TL adapts a model trained on one task to a related task, thereby reducing the need for large amounts of labeled data and accelerating training by leveraging pre-trained representations. ADA uses adversarial training to align feature distributions between source and target domains, making the model more robust to domain shifts. Cross-domain autoencoders learn unified latent representations from different domains, enabling effective knowledge transfer. Re-calibration dynamically adjusts internal feature representations and output probabilities by re-training the model with estimated values, thus compensating for changes in EMG amplitude caused by muscle fatigue and ensuring consistently high performance even after prolonged use.

Electrode shift refers to the reduction in predictive performance that occurs when a model is evaluated with electrode positions different from those used during training owing to the variation in EMG amplitude with electrode placement. Young et al. [23] reported a performance discrepancy of 15% (seven-class scenario) between no-shift and 2 cm shift conditions. Gao et al. [24] reported a performance discrepancy of  20% (six-class scenario) between the no-shift and five-shift conditions of 0, ±1, ±2 cm. In electrode shift scenarios, both TL and ADA have been shown to reduce performance discrepancies. Ameri et al. [13] reported a 7% classification error (eight-class scenario) under shifted conditions, compared with 6% under no-shift conditions (a difference of −1%) using TL with a CNN. Further, Côté-Allard et al. [16] reported a 75.50% classification accuracy (eleven-class scenario) under shifted conditions, compared with 93.58% under no-shift conditions (a difference of −18.08%) using ADA with the Spectrogram ConvNet. Nevertheless, TL requires collecting new data and re-training the DNN model prior to the use of an EMG application. Such frequent calibration is burdensome and discourages users. Moreover, ADA demands data acquired under multiple conditions and recorded at multiple positions, with the electrode shifted 1–3 cm upward, downward, leftward, and rightward to train the model, resulting in lengthy recording sessions that further diminish user acceptance. Thus, a major drawback of these techniques is the reliance on additional data for re-training (TL) or initial training (ADA), which limits their practical deployment. An alternative method that achieves robustness without extra data is therefore required.

To improve cross-subject generalization, we propose sliding-window normalization (SWN) [25], which combines sliding-window processing and z-score normalization. Although the desired improvement in cross-subject generalization was not fully achieved, SWN increased the classification accuracy from 56.2% to 77.7% (a 21.5% improvement) for the same-subject model and from 41.1% to 63.1% (a 21.6% improvement) for models of other subjects in the three-class motion classification. Crucially, SWN achieved these improvements without the need for any additional calibration or training data. SWN improves performance by aligning the EMG amplitude within sliding windows and reducing variability, thereby enhancing signal consistency. Because an electrode shift alters the EMG amplitude due to changes in electrode position [26,27], SWN may mitigate its effects. Unlike TL, ADA, and cross-domain autoencoder approaches, which require extra data, SWN maintains a robust performance solely through its normalization process without extra data. This data efficiency enables SWN to operate effectively in real-world settings, addressing a major limitation of the current methods. Furthermore, SWN can be easily integrated with DNN approaches as a normalization technique. Together, these advantages underscore the practical contribution of SWN in enhancing EMG-based applications for real-world deployment.

Previous studies [13,16] have relied on additional data to achieve performance improvements, limiting their practical use. In contrast, the primary advantage of SWN is that it achieves comparable or improved performance without requiring any additional data. The current study aimed to investigate the effect of SWN on reducing EMG-based real-time classification performance discrepancies while eliminating the requirement for additional data. In Section 3.2, we compare classification accuracy under electrode shift using a CNN-LSTM model for three motion classes with SWN against approaches using TL, ADA, and a mixture of multiple electrode positions’ data (MIX). In Section 3.3, we evaluated the benefits of integrating SWN with these DNN strategies.

## 2. Methods

We acquired three kinds of electrode position data through right-arm continuous motion tasks in a subject experiment (Section 2.1) to investigate whether predictive performance improvement at electrode positions differed from that for training. The measured EMG and body part positions were processed via EMG processing (Section 2.2) and motion label processing (Section 2.3) and used to train the DNN model with a defined training setting (Section 2.4). SWN and DNN strategies (Section 2.5) are compared under the Data Acquisition, Evaluation Index, and Statistical Analysis subsections (Section 2.6).

### 2.1. Data Acquisition

#### 2.1.1. Subjects

The Ethics Board of Nagaoka University of Technology approved this study in accordance with the Declaration of Helsinki (approval no. 2023-03-03). Seventeen right-handed men aged 21–24 years participated in the experiment after being fully informed and providing their consent.

#### 2.1.2. Experiment

**Figure 1:**
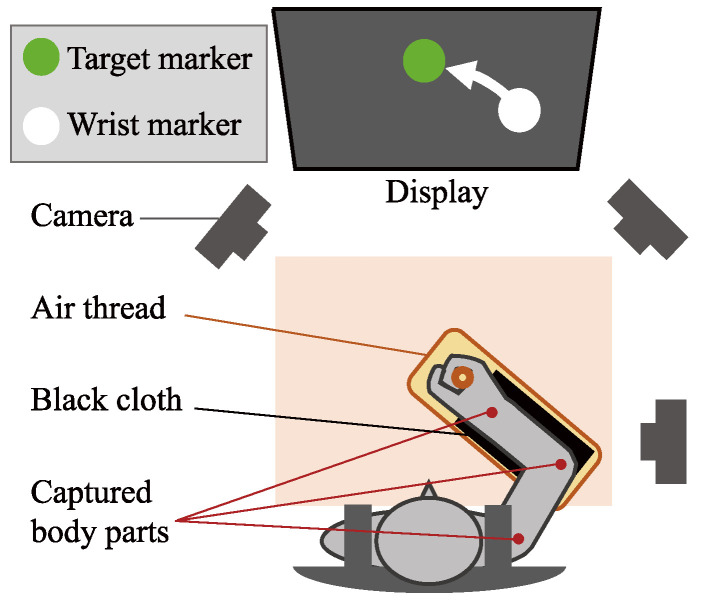
An experimental environment. A black cloth was placed between the air thread and the subject’s right forearm to enhance the detection accuracy of the wrist and elbow joint positions.

In the experiment, data were collected under conditions with shifted electrode positions to evaluate SWN. Subjects performed a target marker-tracking task with their right arm under three electrode position settings (left, center, and right) (Figure 1). The task involved five types of right arm movements (described in Section S4 in the Supplementary Materials) that elicited various motor dynamics (fast, slow, transitional, repeat, and single joint) to evaluate the classification performance. The task was conducted as 60 s trials repeated four times per session (a total of 60 trials in three sessions). Data were measured for each trial. During the experiment, the positions of the right wrist, elbow, and shoulder, as well as the EMG signals from the muscles in the right forearm and right upper arm, were recorded.

The positions of the right wrist, elbow, and shoulder were captured using a sensorless motion capture system (described in Section S3 in the Supplementary Materials; sampling rate: 20 Hz). The system comprised three cameras (Cybershot RX100VII, Sony, Tokyo, Japan), a video capture board (CAM LINK PRO, Elgato, Fremont, CA, USA), and a GPU (NVIDIA GeForce RTX 3070 Ti, NVIDIA, Santa Clara, CA, USA). A black cloth was placed between the air thread and the subject’s right forearm to enhance the detection accuracy of the wrist and elbow joint positions.

The EMG signals were recorded from 12 sites using the Trigno Lab Avanti system (Delsys, Natick, MA, USA, sampling rate: 2000 Hz). The recording sites included the biceps brachii (×4), brachialis (×1), brachioradialis (×1), anconeus (×1), triceps brachii (lateral head) (×2), triceps brachii (long head) (×2), and extensor carpi radialis longus (×1). To thoroughly investigate the impact of electrode displacement on EMG signal acquisition and classification performance, electrode positions were systematically varied across three sessions. In the first session, the electrode was placed at the center at maximal EMG amplitude. In the second session, the electrode was shifted 2 cm to the right of the center perpendicular to the arm’s longitudinal axis. In the third session, it was shifted 2 cm to the left of the center [13]. These controlled shifts in electrode placement allowed for a detailed evaluation of how varying electrode positions affect classification performance.

### 2.2. EMG Processing

**Figure 2:**
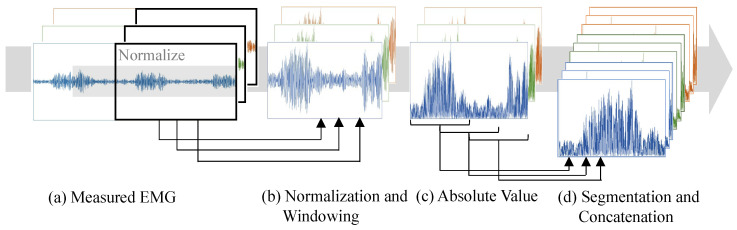
EMG processing to obtain DNN inputs for real-time motion prediction.

For the DNN input, segmented absolute EMG signals were used to efficiently capture temporal variations. The measured data were partitioned on a per-trial basis, thereby eliminating any concerns regarding data leakage. Initially, a 6th-order band-pass Butterworth filter (40–200 Hz) and decimation (2000 → 500 Hz) were applied, followed by normalization over a fixed window length. Subsequently, signals of the desired feature extraction length were obtained, and their absolute values were calculated. The signals were then segmented using a 100 ms window with a 50 ms overlap. Finally, the segmented EMG signals were concatenated across channels (Figure 2). These segmented EMG signals were decimated from 500 to 20 Hz to match the sampling rate of the sensorless motion capture system and were fed continuously into the DNN model. This process ensured that the temporal length of the DNN input remained constant while the number of channels varied according to the EMG length. The butter function and sosfilt function from scipy.signal in Python 3.12 were used for implementation.

### 2.3. Motion Labels Processing

Motion labels (rest, flexion, and extension) were obtained for the elbow joints’ angular velocity and angular acceleration. As an initial verification, we decided to predict a simple motion. Detailed information on this process is provided in Section S5 in the Supplementary Materials.

### 2.4. DNN Model

**Figure 3:**
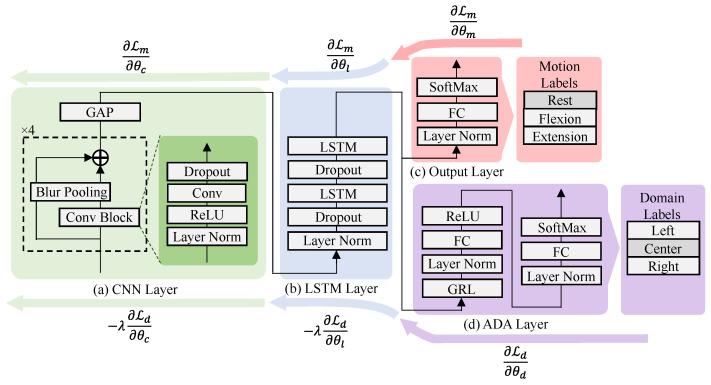
The DNN model: (**a**) CNN layer to extract temporal and channel-wise features, (**b**) LSTM layer to extract temporal-related features, (**c**) output layer to predict motion labels, (**d**) ADA layer to predict electrode positions: left, center, and right (domain labels) for ADA. $L_{m}$ is lost function for motion classifier, whereas $L_{d}$ is lost function for domain classifier. $\theta_{c}$, $\theta_{l}$, $\theta_{m}$, $\theta_{d}$ are the weight parameters for CNN, LSTM, output, and ADA layers. λ is a parameter to control the gradient reversal strength.

We employed a CNN-LSTM model [10] as our DNN architecture (Figure 3), which was capable of extracting local spatiotemporal features via CNN layers and capturing temporal evolution via the LSTM layer. This model predicted three classes: rest, flexion, and extension of the elbow. The model comprised four components: a CNN layer, an LSTM layer, an output layer, and an ADA layer. The CNN layer consisted of four convolutional blocks, followed by Blur Pooling [28] and Global Average Pooling (GAP). Each convolutional block included a temporal layer normalization, a ReLU activation, a 1D convolution (with output channel size equal to the input channel size, kernel size: 3, stride: 1, padding: 2), and dropout with rates of 0.1, 0.2, 0.3, and 0.4 for successive blocks. Blur Pooling (kernel size: 3, stride: 2, and padding: 1 applied only in the second block) was used to reduce aliasing effects during downsampling. The LSTM layer applied layer normalization, followed by two LSTM modules (with input and hidden sizes equal to the number of input channels) and dropouts (rate of 0.1). The output layer included layer normalization, a fully connected module (with an output size equal to the number of input channels), and the softmax function. When ADA is performed, the ADA layer is activated. The ADA layer includes a Gradient Reversal Layer (GRL, with λ=1.0; a parameter to control the gradient reversal strength), two-layer normalization layers, two fully connected modules (with an output size equal to the number of input channels), rectified linear unit activation, and softmax function. It was used solely to predict the electrode positions: left, center, and right. Focal Loss [29] was used as the loss function in (1) because it facilitates training with unbalanced class labels.

(1) $$\begin{array}{c} L^{F}\left(y,\hat{y}\right)=\sum_{n=1}^{N}\alpha_{l}\;{\left(1-p_{n}\right)}^{\gamma }\;L^{\mathrm{CE}}\left(y_{n},{\hat{y}}_{n}\right),\\ \alpha_{l}=\frac{\mathrm{count}{\left(y\right)}_{l}}{\mathrm{count}(y)},\\ p_{n}=\exp\left(-L^{\mathrm{CE}}\left(y_{n},{\hat{y}}_{n}\right)\right). \end{array}$$

Here, $\alpha_{l}$ denotes the label rate for label *l*, γ is a parameter that adjusts $\left(1-p_{n}\right)$, $L^{\mathrm{CE}}\left(y_{n},{\hat{y}}_{n}\right)$ represents the cross-entropy loss between the *nth* true label $y_{n}$ and the *nth* predicted label ${\hat{y}}_{n}$, and count(·) is a count function that returns the number of elements in its argument; specifically, $\mathrm{count}{\left(y\right)}_{l}$ denotes the number of true labels equal to *l*, and count(y) denotes the total number of labels. The DNN model was implemented using PyTorch 2.4.1 and PyTorch Lightning 2.4.0.

### 2.5. Comparison Methods

In this study, we first compared the classification performance achieved using SWN as a normalization method against other approaches—namely, transfer learning (TL), adversarial domain adaptation (ADA), and a mixture of multiple electrode positions’ data (MIX) as described in Section 3.2. Furthermore, as SWN is a normalization method that can be expressed in combination with other techniques, we evaluated its integration with the TL, ADA, and MIX approaches.

#### 2.5.1. Sliding-Window Normalization (SWN)

SWN is a real-time technique that integrates sliding-window processing with z-score normalization and is applied before feature extraction (Figure 2). It was originally proposed to attenuate inter-subject variability in EMG signals [25]. Our previous study [25] showed that SWN generally improves the classification accuracy in both the subject-specific and cross-subject models, although it does not always reduce inter-subject amplitude variability. We speculate that this benefit arises at least in part from diminishing inter-channel amplitude disparities by aligning EMG amplitudes. Navallas et al. [30] demonstrated that amplitude scaling increased EMG probability density function (PDF) and aligned the EMG filling curve across signals with different amplitudes, implying that common EMG analyses become feasible once amplitude differences are compensated through amplitude normalization. Regarding electrode shifts, we assume that displacements of only a few centimeters introduce minimal amplitude changes and that subsequent amplitude alignment can, therefore, improve classification performance. The SWN processing is defined in Equation (2):

(2) $$y_{t,\;n-t+L^{\mathrm{norm}}}=\frac{x_{n}-m_{t}}{s_{t}}\;\left(t-L^{\mathrm{norm}}<n\leq t\right)$$

Here, *t* represents the current discrete time, $L^{\mathrm{norm}}$ is the sliding-window length, *n* is the discrete-time index within the sliding window, $x_{n}$ denotes the *n*th processed EMG value, and $y_{t,\;n-t+L^{\mathrm{norm}}}$ is the EMG signal after applying SWN corresponding to the $n-t+L^{\mathrm{norm}}$th element at time *t*. Parameters $m_{t}$ and $s_{t}$ represent the mean and standard deviation computed over the sliding window at time *t*, respectively. The mean and std functions in NumPy were used in Python to compute these statistics.

#### 2.5.2. Vanilla

The term “Vanilla” denotes the plain model without any enhancements from TL, MIX, or ADA. In this case, the model comprised only an unmodified CNN-LSTM.

#### 2.5.3. Transfer Learning (TL)

TL is a technique that leverages knowledge from a pre-trained model by initially training on a large source domain dataset and then freezing the feature extraction layers while re-training the output layers with a limited amount of target domain data. We selected TL because it is one of the most effective methods for improving classification performance when adapted to shifted-electrode data [13]. In this study, we froze only the CNN layer and retrained the LSTM and output layers because time-related features captured by the LSTM may be affected by changes in electrode position.

#### 2.5.4. Adversarial Domain Adaptation (ADA)

ADA employs two output layers: one for class label classification and another for domain label classification. Typically, ADA is trained in an unsupervised manner using source domain data with class labels and target domain data without class labels, with a domain classifier that distinguishes source from target domains [16]. However, in this study, we employed supervised adversarial multi-domain adaptation [12]. In our approach, the ADA was trained on mixed data, including all three electrode positions (left, center, and right), and the domain classifier was designed to predict the specific electrode position rather than simply distinguishing the source from the target domains. This strategy enabled the ADA model to capture the diversity present in multi-condition data. ADA was selected for comparison with the proposed SWN because SWN does not require re-training when the electrodes are shifted.

#### 2.5.5. Mixture of Multiple Electrode Positions’ Data (MIX)

The MIX approach is similar to ADA but does not employ a multi-domain classifier. In this method, the models are trained using mixed data from all three electrode positions (left, center, and right) [13]. We adopted MIX for comparison with ADA and SWN because it does not require re-training when the electrodes are shifted.

### 2.6. Training and Evaluation Criteria

#### 2.6.1. Training, Tuning, and Testing Data

The training, tuning, and testing datasets were defined according to the DNN strategy, as detailed in Table 1. In this study, the sizes of the training and testing data were each kept nearly the same across all DNN strategies. In the Vanilla and TL models, 70% of data from a single electrode position were randomly drawn for training. In the TL, a tuning dataset was acquired from 30% of the common training dataset as Vanilla and TL, which was the same electrode position as the testing dataset. For the ADA and MIX models, the training dataset contained 30% of the data from each of the three electrode positions sampled from the common training dataset shared with Vanilla and TL. For all four models, 30% of the data from the selected electrode positions were reserved as a common testing set, and the same testing samples were used across the models to guarantee a fair comparison. The training epochs, batch size, time length for training data, and optimizer settings were identical across TL, MIX, ADA, and Vanilla models: 40 training epochs, a batch size of 128, time length for training data of 20 s, and the Adam optimizer (learning rate: $1.0\times 10^{-3}$ in training and $1.0\times 10^{-4}$ in re-training, $\beta_{1}=0.9$, and $\beta_{2}=0.999$). In addition, for the TL, we performed 10 re-training epochs for one of the different electrode positions.

#### 2.6.2. Testing Method

In this section, we describe the selection process for the comparison results. For the TL and Vanilla models, training and testing data were obtained from different electrode positions (yielding six combinations). Training data were collected for the MIX and ADA models from all three electrode positions, while testing data were selected from one electrode position (yielding three combinations). The best classification performance was determined by computing the mean classification accuracy for each electrode position combination and subject, and by selecting the maximum mean performance over the range of window length parameters. For the no-normalization (None) case, the optimal classification performance was selected based on feature-extraction window lengths (ranging from 200 to 1000 ms in 200 ms intervals). For SWN, the optimal classification performance was selected from among Various combinations of normalization window lengths (200–1000 ms in 200 ms intervals) and feature extraction window lengths (200–1000 ms in 200 ms intervals).

#### 2.6.3. Evaluation Index

We used the differential classification accuracy as an evaluation metric to assess the reduction in the impact of electrode shift on classification performance. As defined in Equation (3), this metric was calculated for each subject and for each combination of training and testing. electrode positions. The differential classification accuracy was computed by subtracting the accuracy of BASELINE (obtained using the same electrode position for the training and testing data and the same normalization method) from the accuracy achieved by each DNN strategy.

(3) $$\begin{array}{c} y_{n,i,j}=x_{n,i,j}-x_{n,j,j}^{\mathrm{BASELINE}}\\ x=\frac{\mathrm{Success}\;\mathrm{of}\;\mathrm{Predictions}}{\mathrm{Success}\;\mathrm{of}\;\mathrm{Predictions}+\mathrm{Failure}\;\mathrm{of}\;\mathrm{Predictions}} \end{array}$$

where *n* denotes the normalization method, *i* refers to the training electrode position data, and *j* corresponds to the tuning and testing of the electrode position data. The variable $y_{n,i,j}$ represents the differential classification accuracy using the training data from electrode position *i* and the tuning and testing data from electrode position *j* under the normalization method *n*. The terms $x_{n,i,j}$ denote the classification accuracy obtained using the training data from electrode position *i* and tuning and testing data from electrode position *j*, while $x_{n,j,j}^{\mathrm{BASELINE}}$ represents the best classification accuracy obtained using the same electrode position for both training and testing, based on the optimal window lengths for normalization and feature extraction (BASELINE, as described in Table 1).

#### 2.6.4. Statistical Analysis

Statistical significance was evaluated using the Wilcoxon rank-sum test with a significance level of p<0.05, applying the Bonferroni correction for multiple comparisons. We calculated the mean differential classification accuracy across the electrode position combinations for each subject and used these averages to test for statistical significance. The ranksums function from scipy.stats and the multipletests function from statsmodels.sandbox.stats.multicomp in Python were used for this purpose. Additionally a two-way Scheirer–Ray–Hare test was used for between-group comparisons between SWN and no-normalization condition across TL, ADA, and MIX using scheirerRayHare function from rcompanion and the Formula function from robjects in rpy2 (R-4.4.3).

## 3. Results

We examined the impact of varying window lengths for normalization and feature extraction (Section 3.1) and then evaluated the performance of SWN and its integration using the DNN strategies (Section 3.2 and Section 3.3). _SWN means with SWN, and _None means no normalization. All classification results were compared at a chance level of 33.3% (three classes: rest, flexion, and extension).

### 3.1. Effects of Window Lengths

**Figure 4:**
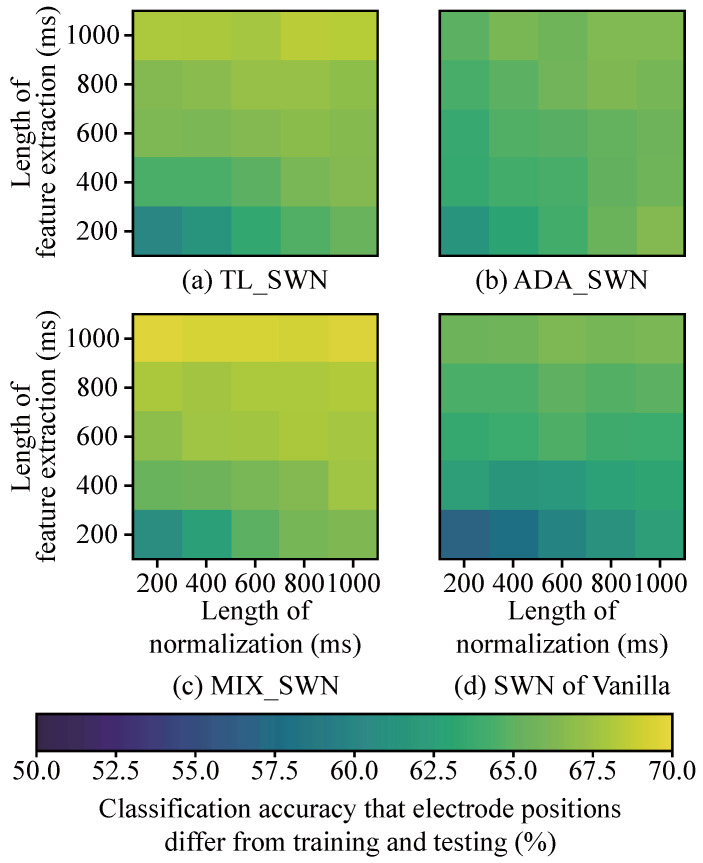
The effects of window length on feature extraction with SWN. (**a**–**d**) indicate DNN strategies. The color indicates the classification accuracy.

**Figure 5:**
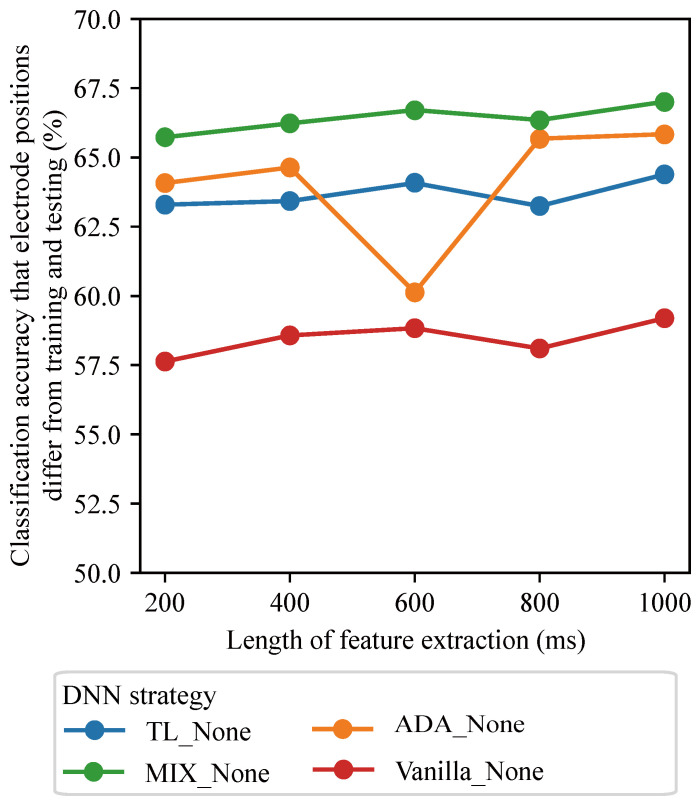
The effects of window length on feature extraction without normalization.

We investigated how different window lengths for normalization and feature extraction affected classification accuracy across various DNN strategies and electrode position combinations. Figure 4 and Figure 5 present the results obtained when the window lengths were varied. For SWN (Figure 4), both the normalization and feature extraction window lengths were varied from 200 to 1000 ms in 200 ms increments. Under the no-normalization condition (Figure 5), only the feature extraction window lengths were varied over the same range. We defined the range of window lengths for normalization and feature extraction to be between 200 and 1000 ms, owing to its limited calculation time. Therefore, most studies have used up a 1000 ms window length [31,32,33].

In Figure 4a–d, increasing the window lengths for both normalization and feature extraction consistently improved the accuracy. In particular, Figure 4a,c,d indicate that using a 1000 ms window for normalization enhances classification accuracy regardless of the feature extraction window length. The best performance in Figure 4 was achieved using MIX_SWN, with a classification accuracy of 69.6%. Based on these results, we recommend selecting a 1000 ms window for both normalization and feature extraction so that the window length maximizing accuracy can be chosen. The optimal window lengths were determined as follows: 600 ms for normalization and 1000 ms for feature extraction in the SWN of Vanilla, 800 ms and 1000 ms in TL_SWN, 1000 ms and 200 ms in ADA_SWN, and 200 ms and 1000 ms in MIX_SWN.

Similarly, in Figure 5 increasing the feature extraction window length improved accuracy. MIX_None achieved the best result (67.0% accuracy) with a 1000 ms window for feature extraction. This indicates that a 1000 ms window is optimal for feature extraction under the no-normalization condition. In Vanilla_None, TL_None, ADA_None, MIX_None, and BASELINE_None, the optimal window length for feature extraction was 1000 ms.

### 3.2. Comparison of Alternative Methods Against SWN

**Figure 6:**
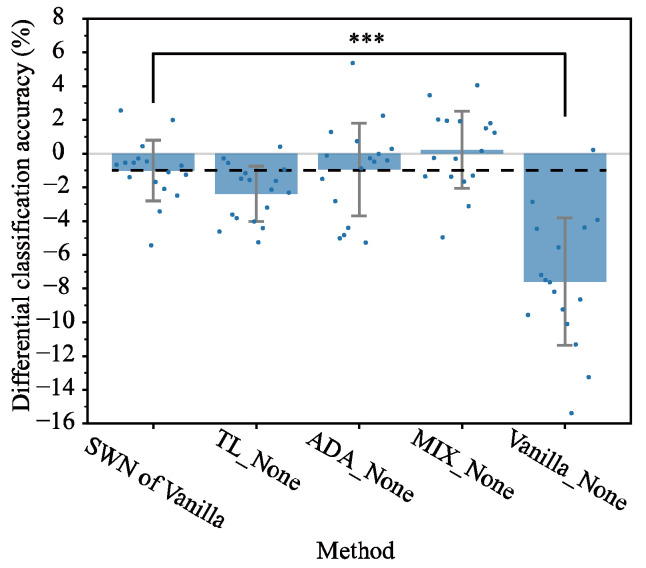
Performance comparison between SWN and other compared methods. None indicates no normalization. The blue dot markers in a blue bar indicate mean differential classification accuracy computed among electrode position combinations for each subject, the error bar represents the standard deviation across subjects, the black dashed line indicates the differential classification accuracy for SWN of Vanilla, and the gray line indicates 0%. *** means p<0.001.

To assess whether SWN improves classification accuracy under electrode shift conditions, we compared the SWN of Vanilla with TL_None, ADA_None, MIX_None, and Vanilla_None (Figure 6). In SWN of Vanilla, the optimal BASELINE window lengths were 1000 ms for normalization and 600 ms for feature extraction. In contrast, for TL_None, ADA_None, MIX_None, and Vanilla_None, the optimal BASELINE window length for feature extraction was 1000 ms.

As shown in Figure 6, MIX_None achieved the highest performance with a difference of 0.2% in classification accuracy, which is 1.2% higher than SWN of Vanilla; however, this was not statistically significant (p>0.05). SWN of Vanilla tied with ADA_None (both at −1.0%) and outperformed TL_None by 1.4% (p>0.05) and Vanilla_None by 6.6% (p<0.001). These results indicate that SWN enhances classification accuracy across different electrode positions without requiring additional data, outperforming TL (which depends on the extra electrode position data) and achieving comparable performance with ADA and MIX.

### 3.3. Comparison of DNN Methods with SWN Integration

**Figure 7:**
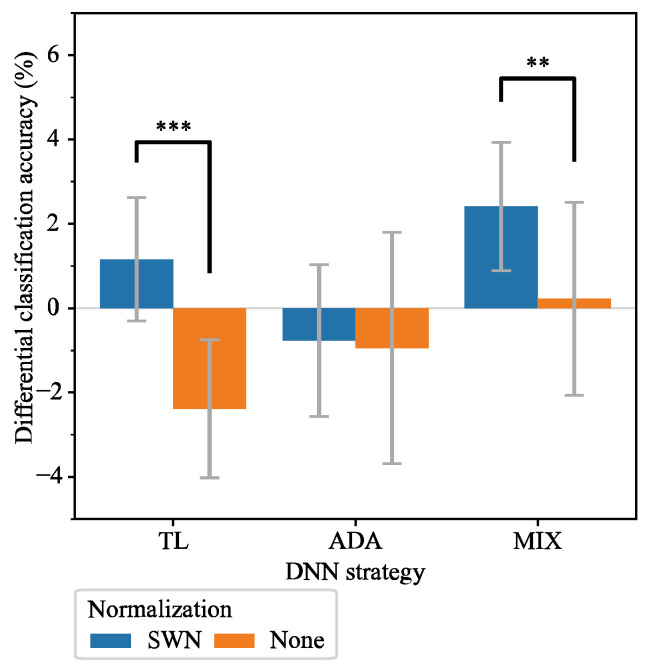
Performance comparison between DNN strategies with SWN and without normalization. None indicates no normalization. The error bar represents the standard deviation across subjects of the mean differential classification accuracy computed among electrode position combinations, and the gray line indicates 0%. ** means p<0.01, and *** means p<0.001.

**Figure 8:**
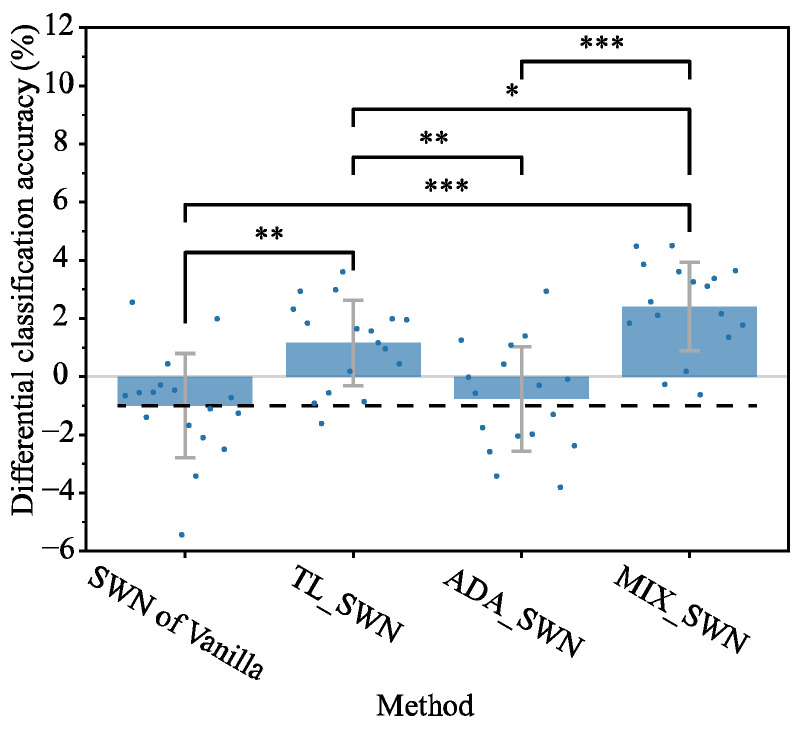
Performance comparison among DNN strategies with SWN integration. The blue dot markers in a blue bar indicate mean differential classification accuracy computed among electrode position combinations for each subject; the error bar represents the standard deviation across subjects, the black dashed line indicates the result of SWN of Vanilla, and the gray line indicates 0%. * means p<0.05, ** means p<0.01, and *** means p<0.001.

In Section 3.2, we demonstrated that SWN improves classification accuracy when using the Vanilla model without additional electrode position data. Here, we investigated whether integrating SWN with various DNN strategies further enhances classification accuracy. Figure 7 compares DNN strategies with SWN against their no-normalized counterparts. Furthermore, Figure 8 compares the SWN of Vanilla with other DNN strategies integrated with SWN. In SWN integrations, the optimal BASELINE window lengths were identical to those obtained for SWN of Vanilla in Section 3.2, whereas, in the no-normalized methods, the optimal window length for feature extraction remained as described in Section 3.2.

Figure 7 shows that applying SWN significantly improved the difference in classification accuracies for TL and MIX. A two-way Scheirer–Ray–Hare test revealed highly significant difference between SWN and no normalization ($p=8.70\times 10^{-10}$), indicating that the effect of SWN is consistent across methods. Specifically, TL_SWN achieved a 1.2% difference, which was 3.6% higher than TL_None (−2.4%, p<0.001), and MIX_SWN achieved a 2.4% difference, which was 2.2% higher than that of MIX_None (0.2%; p<0.01). In contrast, ADA_SWN (−0.8%) was not significantly different from ADA_None (−0.9%, p>0.05). These results indicate that integrating SWN with DNN strategies further enhances the classification accuracy under electrode shift conditions.

Figure 8 clearly shows that SWN integrations significantly improve classification accuracy compared with the SWN of Vanilla. Among SWN-integrated methods, MIX_SWN achieved the greatest improvement of 2.4%, followed by TL_SWN, with a 1.2% increase. Moreover, MIX_SWN outperformed TL_SWN by 1.3% (p<0.05), and when compared with SWN of Vanilla, TL_SWN and MIX_SWN showed improvements of 2.2% and 3.4%, respectively. TL_SWN and MIX_SWN surpassed BASELINE (0%) by 1.3% and 2.4%, respectively.

## 4. Discussion

In this study, we applied SWN to mitigate the reduction in classification accuracy due to electrode shift. We improved the electrode shift issue, as applying SWN without additional data enhanced the differential classification accuracy to −1.0%—an improvement of 6.6% (Figure 6). Although this requires additional data, the MIX strategy exceeded classification accuracy relative to the no-shift condition at 2.4%. It also eliminated—and even reversed—the accuracy deficit, yielding classification results that exceed those obtained under the no-shift condition. We succeeded in improving the differential classification accuracy from −7.6% to −1.0%—an improvement of 6.6%—by applying SWN (Figure 6). These results demonstrate the effectiveness of SWN.

In this section, we discuss (1) the performance of the SWN of Vanilla compared with alternative DNN strategies without normalization (Section 4.1); (2) the performance of DNN strategies integrated with SWN (Section 4.2); (3) selection of parameters for SWN (Section 4.3); and (4) the strengths and limitations of SWN (Section 4.4).

### 4.1. Performance of SWN

SWN mitigated the reduction in classification accuracy due to electrode shift; in particular, SWN of Vanilla (−1.0%) achieved a 6.6% improvement in differential classification accuracy compared with Vanilla_None (−7.6%) (Figure 6 in Section 3.2). Moreover, its performance was nearly equivalent to that of ADA_None (−0.9%) and 1.4% higher than TL_None (−2.4%). However, the SWN of Vanilla was 1.2% lower than that of MIX_None (0.2%). Although the performance of the SWN of Vanilla was not the highest, SWN notably enhanced the results using data from only a single electrode position, whereas other DNN strategies required data from multiple electrode positions. This incremental improvement is attributable to the fact that our method does not require any additional data, thereby significantly reducing the calibration and re-training efforts and costs in practical applications, which is extremely advantageous in real-world applications. Furthermore, our method can readily integrate with the latest DNN architectures and be applied to more complex multi-class motion prediction problems; further performance enhancements can be anticipated, suggesting a promising avenue for future research and practical deployment.

Surprisingly, the best performance was achieved with MIX_None (Figure 6), which simply combines data from multiple electrode positions without employing ADA or TL. This higher performance compared with BASELINE_None (Table 1) is likely because the model was trained on a mixture of data from multiple electrode positions, enabling them to adapt to various environments. This phenomenon was also observed in a previous study on cross-subject generalization (i.e., the model trained using data from other subjects) [25], where the number of subjects improved classification accuracy. Although we initially expected ADA_None to outperform MIX_None, MIX_None achieved the best performance. These findings suggest that training with a mixture of multiple conditions is superior to specialized DNN strategies, especially when electrode positions are shifted by approximately 2 cm and multiple datasets with motion labels are available. A shift of approximately 2 cm resulted in minor changes in the amplitude and local features of the EMG signals, whereas the overall pattern remained largely unchanged, making it an ideal condition for capturing and learning subtle variations. In contrast, a previous study [13] (ten-class scenario) reported that TL achieved a 6% classification error, which was 1% lower than the 7% error obtained using the MIX approach—the opposite of our findings. We attributed this discrepancy to data imbalance, as 75% of the training data came from no-shifted electrodes, whereas only 25% came from shifted electrodes. In that study, data from the no-shifted condition were approximately three times more abundant than those from the shifted condition, likely causing the model to become overly adapted to the no-shifted state, resulting in higher accuracy for TL than for MIX. By contrast, in our study, the data were more evenly balanced, and the MIX approach outperformed TL. Thus, the composition of the training data is a crucial factor in the selection of a strategy to mitigate performance degradation. Moreover, the MIX approach is the simplest because it merely involves combining the data from multiple electrode positions.

### 4.2. DNN Strategies with SWN Integration

The TL and MIX models integrated with SWN show improved performance compared with their non-normalized counterparts. Specifically, TL_SWN exhibited 3.6% higher differential classification accuracy than TL_None, and MIX_SWN achieve the best performance with a 2.4% improvement—2.2% higher than MIX_None—as shown in Section 4.1. In contrast, ADA_SWN improved by only 0.2%, showing little difference compared with ADA_None. These results indicate that TL and MIX exhibit a synergistic effect when integrated with SWN. Furthermore, training using a combination of multiple conditions is superior to specialized DNN strategies, particularly when electrode positions are shifted by approximately 2 cm and multiple datasets with motion labels are available. Moreover, we believe that the electrode shift issue can be effectively addressed, as both TL_SWN and MIX_SWN outperformed models trained and tested on data from the same electrode position. To our knowledge, no previous study has demonstrated that a normalization approach can mitigate the issue of electrode shift to the extent observed in our study. In addition, no prior study has combined multiple methods to address electrode shift, nor has any prior study achieved performance that exceeds the baseline. Our results indicate that integrating SWN with DNN strategies (TL or MIX) effectively mitigates the electrode shift issue, outperforming even models trained and tested on data from a single electrode position.

### 4.3. Parameters Selection for Proposed SWN

Figure 4 indicates that longer window lengths for both normalization and feature extraction (200–1000 ms) yielded a higher classification accuracy. Further, a longer window length for feature extraction is more important than one for normalization to increase classification accuracy. Especially in MIX_SWN, classification accuracies at the 1000 ms feature extraction window between 200 and 1000 ms normalization window were 69%, the same predictive performance.

**Figure 9:**
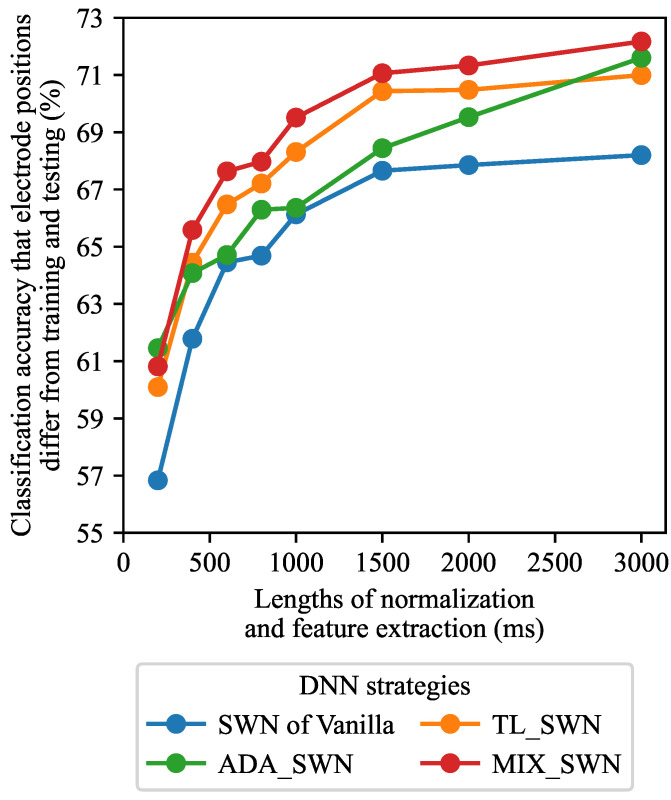
Window length analysis on SWN. The color indicates DNN strategies.

Moreover, we evaluated classification accuracy at 1500, 2000, and 3000 ms window lengths for normalization and feature extraction to investigate weather classification accuracy increases. Figure 9 indicates a classification accuracy comparison among the same window length combinations for normalization and feature extraction. From Figure 9, classification accuracy was best at 3000 ms window lengths for normalization and feature extraction. This result also indicates the longer window lengths for both normalization and feature extraction were the best, as shown in Figure 4. However, in real-time processing, the calculation time is limited. Song et al. [34] reported a 96.6% classification accuracy and a 2 s calculating time in a 2000 ms window length for feature extraction. Previous studies recommended the following: 200–300 ms window length for feature extraction [33]. Therefore, a 3000 ms feature extraction window is not practical, and the shorter window length of less than 1000 ms is better considering both performance and computational cost.

### 4.4. Strengths and Limitations of SWN

The strength of SWN is no need for additional measured data, such as calibration data and other electrode positions’ data, to narrow the classification accuracy gap based on no-shift owing to the electrode shift (Figure 6). Although this requires additional data, the MIX strategy exceeded classification accuracy relative to the no-shift condition (Figure 8). However, the data were acquired with electrodes displaced by 2 cm in the direction perpendicular to the arm; we did not evaluate predictive performance for parallel displacements, smaller offsets such as 1 cm, larger shifts of 3 cm or more, or rotated electrode orientations.

Future work will proceed along four concrete directions: (1) **Diverse electrode positions validation** —We will evaluate the performance of SWN under diverse shifted and rotated electrode position conditions in the electrode shift: The electrodes were shifted by 0, 1, 2, and 3 cm upward, downward, leftward, and rightward, and rotated by $0^{\circ }$, $\pm 20^{\circ }$, $\pm 40^{\circ }$, and $\pm 60^{\circ }$; (2) **Multi-subject, electrode-position, and day validation**—We need to assess SWN robustness to day-to-day factors such as muscle fatigue and perspiration, targeting an across-day coefficient of variation below 5% [16,17,18,25]. We subsequently will evaluate SWN on cross-subject datasets recorded at multiple electrode positions over several days, aiming to limit the accuracy gap between personal models and cross-subject models to within −1.0%; (3) **Expansion of the motion set**—We will add forearm pronation/supination and shoulder flexion/extension to the elbow-centric dataset, yielding the following nine classes: problem from which we will reconstruct 3D joint trajectories and aim for a mean angular error below $10^{\circ }$ [35,36]. (4) **Assessment on trans-radial amputees**—Previous studies reported fine-tuning only the final fully connected layer for 5-min amputee-specific data [37,38]. Therefore, we can improve the degradation of the classification accuracy owing to the electrode shift using amputee-specific data. We aim for at least a 5-percentage-point gain over a no-calibration baseline, building on prior evidence that TL boosts accuracy; and (5) **Extension to regression tasks**—Embed SWN in a temporal convolutional network to predict continuous elbow angle or angular velocity, aiming for over 95% performance via R2 score.

## 5. Conclusions

In this study, we mitigated the degradation of classification accuracy caused by electrode shifts by introducing SWN. The performance was quantified as the differential classification accuracy between each deep-learning strategy (trained at one electrode position and tested on another) and BASELINE (trained and tested at the same electrode position). SWN (Vanilla CNN-LSTM with SWN) narrowed the differential classification accuracy to −1%, a 6.6% improvement compared with the −7.6% baseline without normalization (Vanilla_None). SWN mitigates electrode-shift errors while relying only on data from a single-electrode location for training, thus sparing users from both multi-position data collection and pre-use calibration. Moreover, SWN combined with multiple position training (MIX_SWN) yielded a differential classification accuracy that exceeded the no-shift condition by 2.4%, demonstrating that the electrode shift issue has been resolved.

Future work will concentrate on five directions: (1) diverse electrode position validation, (2) multi-subject, electrode position, and day validation, (3) expansion of the motion set, (4) assessment of trans-radial amputees, and (5) extension to regression tasks.

## Figures and Tables

**Table 1:** The DNN strategy and various data settings. ADA and MIX do not conduct transfer learning.

DNN Strategy	Training Data	Tuning Data	Testing Data
Vanilla, TL	70% of the data acquired from one electrode position	30% of the data acquired in the Vanilla and TL common training dataset from the electrode position employed for testing (Only TL)	30% of the data acquired from one of the different electrode positions from one used for training
ADA, MIX	30% of the data acquired from each of the three electrode positions in Vanilla and TL common training dataset	-	Same common testing dataset as Vanilla and TL from one of the electrode positions
BASELINE	Same common training dataset as Vanilla and TL	-	Same common testing dataset as Vanilla and TL from the electrode position employed for training

## Data Availability

The data are not publicly available due to privacy.

## Supplementary Materials

The following supporting information can be downloaded at: https://www.mdpi.com/article/10.3390/s25134119/s1, refs. [39,40,41] are cited in Supplementary Materials, Section S1: Comparison of Alternative Methods Against the SWN; Section S2: Comparison of DNN methods with the SWN Integration; Section S3: Sensorless Motion Capture System; Section S4: Generated Tasks; Section S5: Motion Labels Processing.
