# Supplementary material for: Mitigating the Impact of Electrode Shift on Classification Performance in Electromyography Applications Using Sliding-Window Normalization
Source: Sensors (Basel). 2025 Jul 1;25(13):4119. doi: 10.3390/s25134119 (PMC12251759; doi:10.3390/s25134119)
Supplement: Supplementary file 1 [file sensors-25-04119-s001.zip › supplementary materials/FiguresMethods/Motion Labels Processing.pdf]

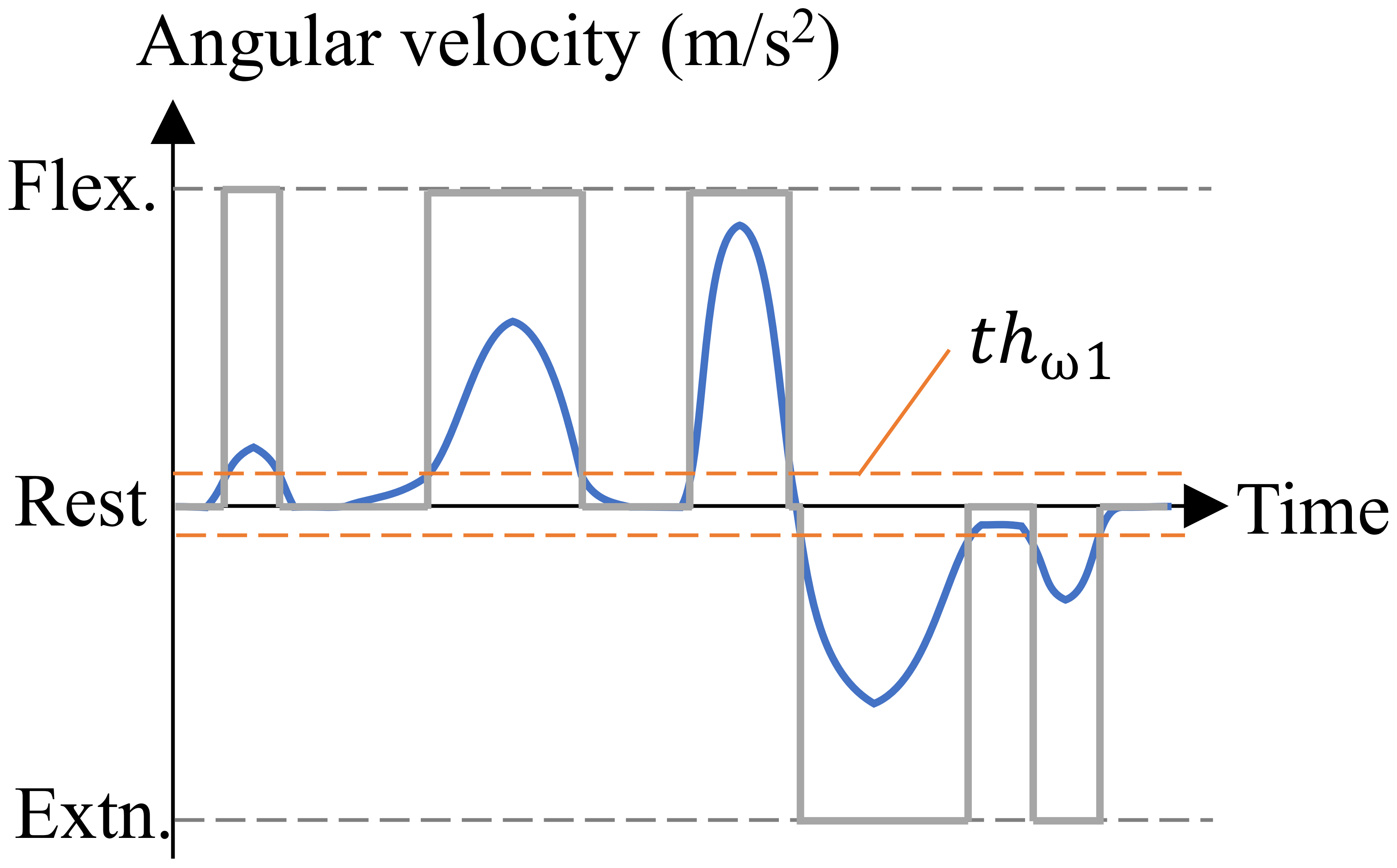

(a) Simple labeling.

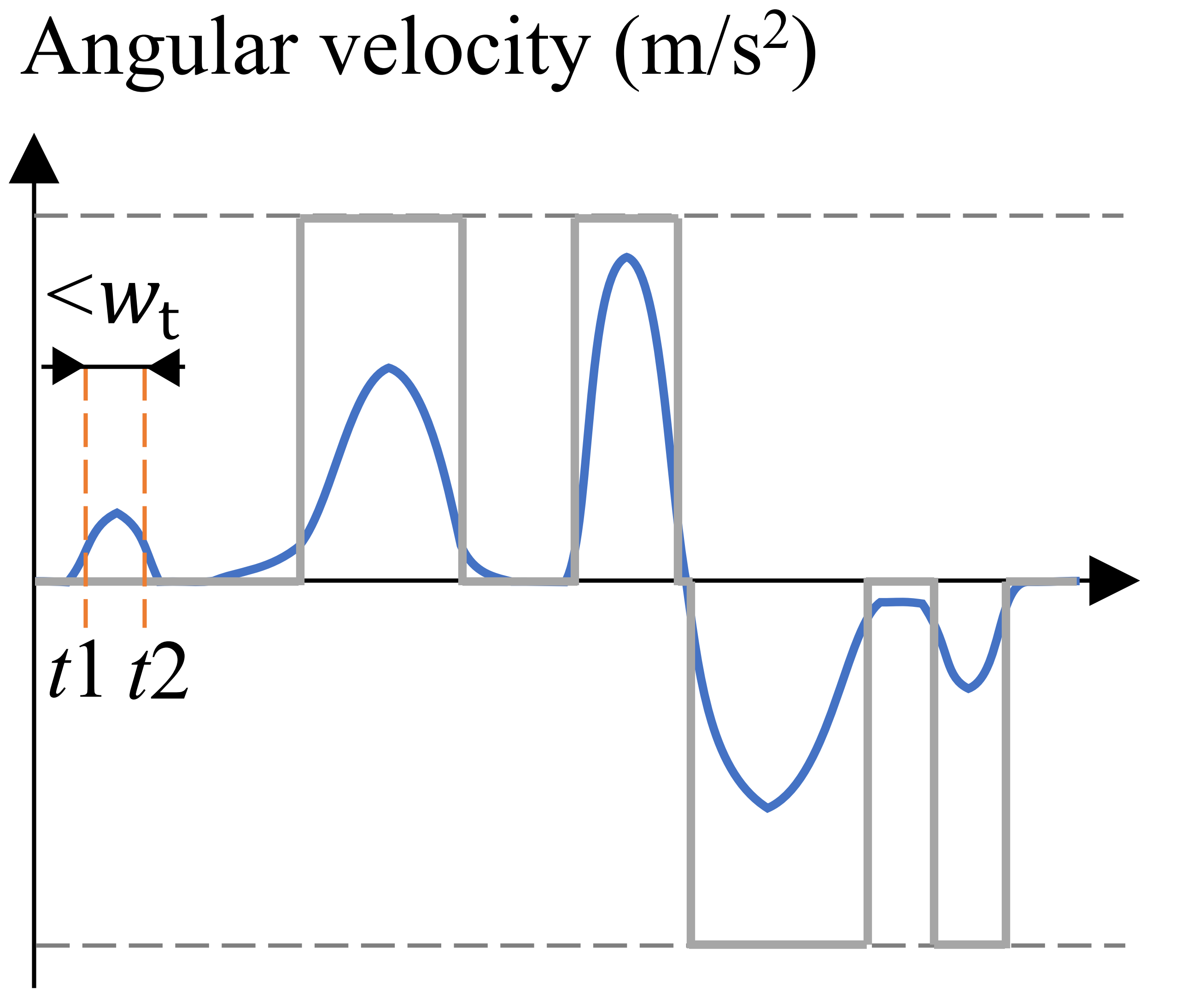

(b) Replacing shot-time movements.

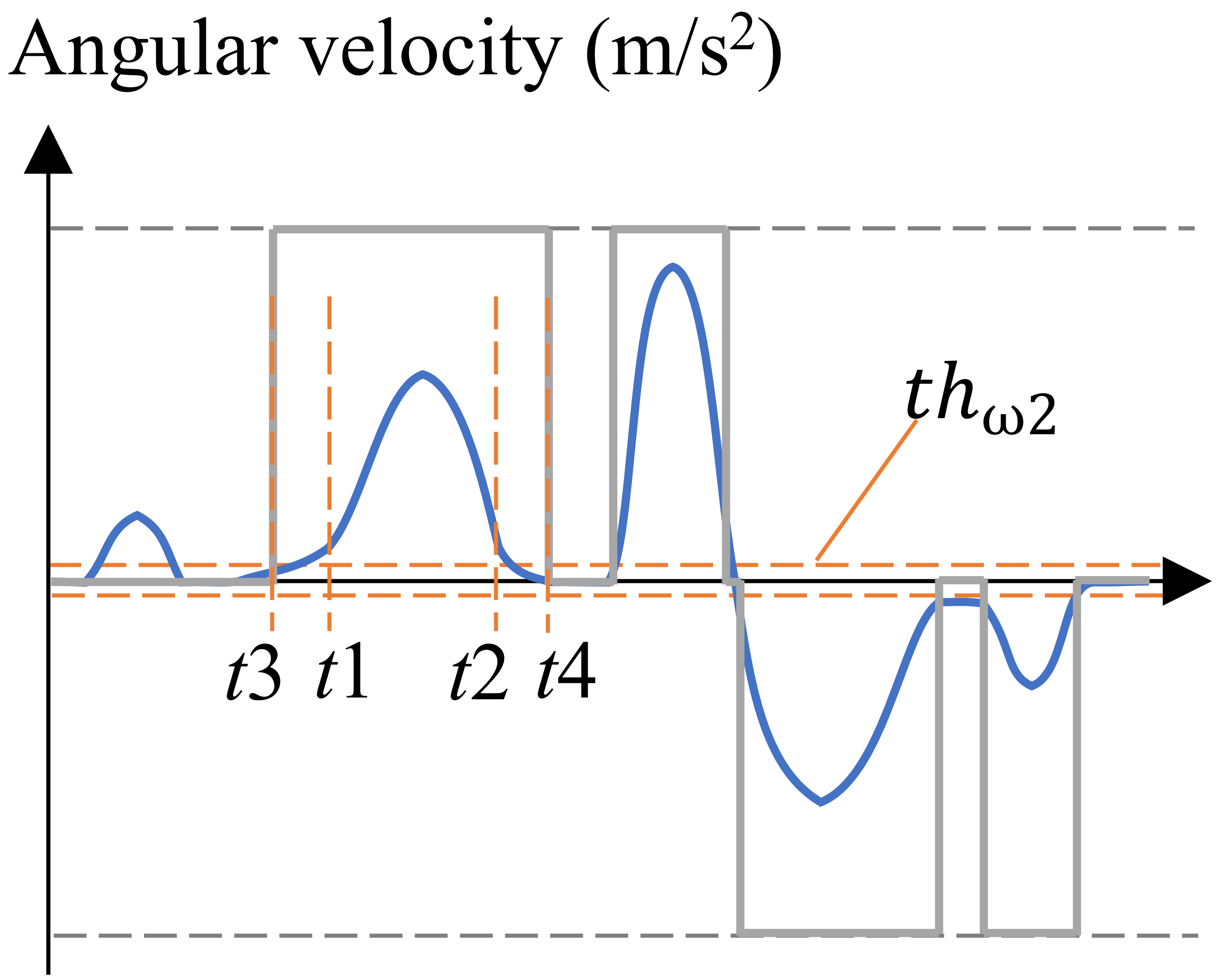

(c) Extending time width of movements.

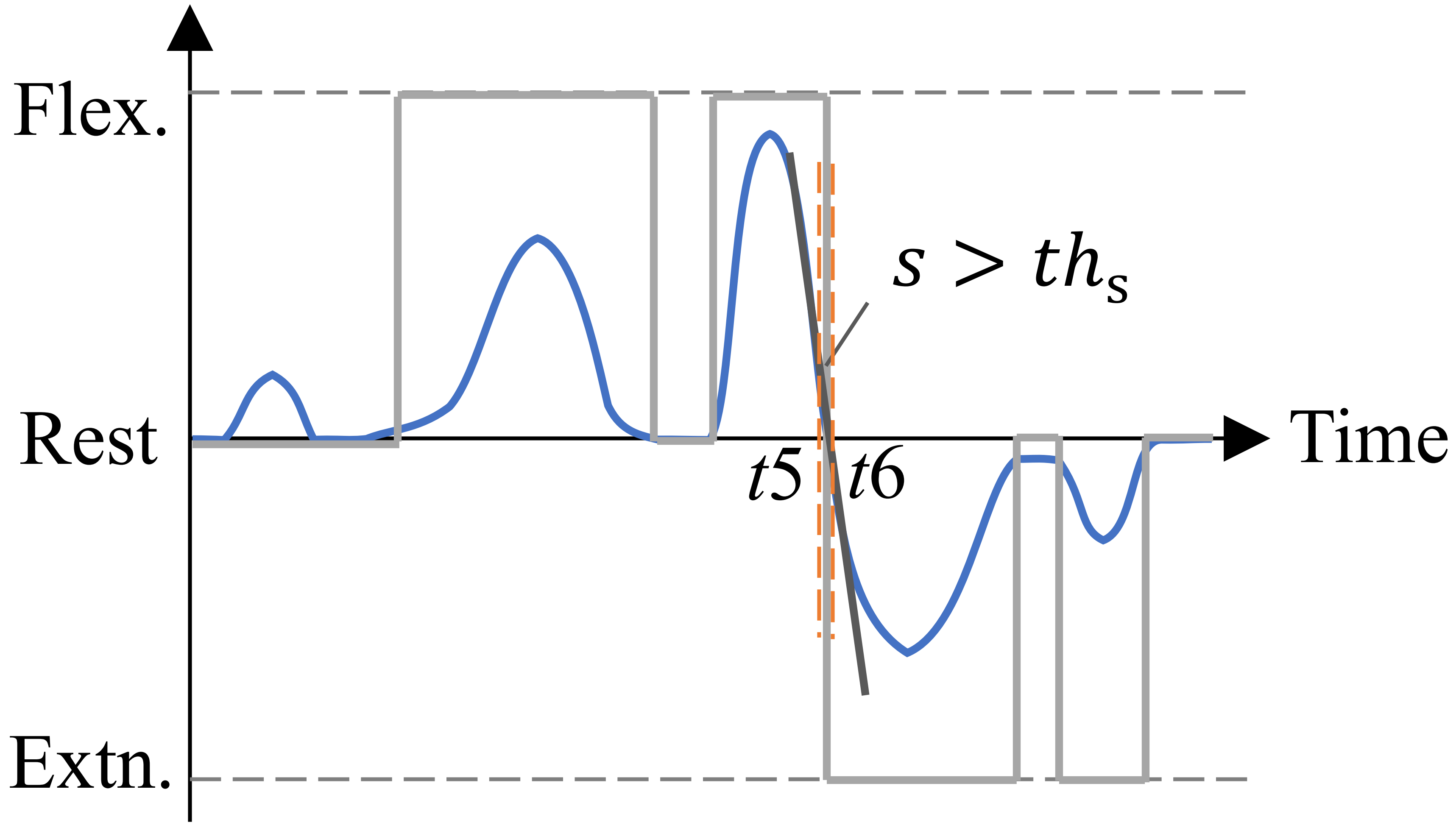

(d) changing rest to continuous movement.

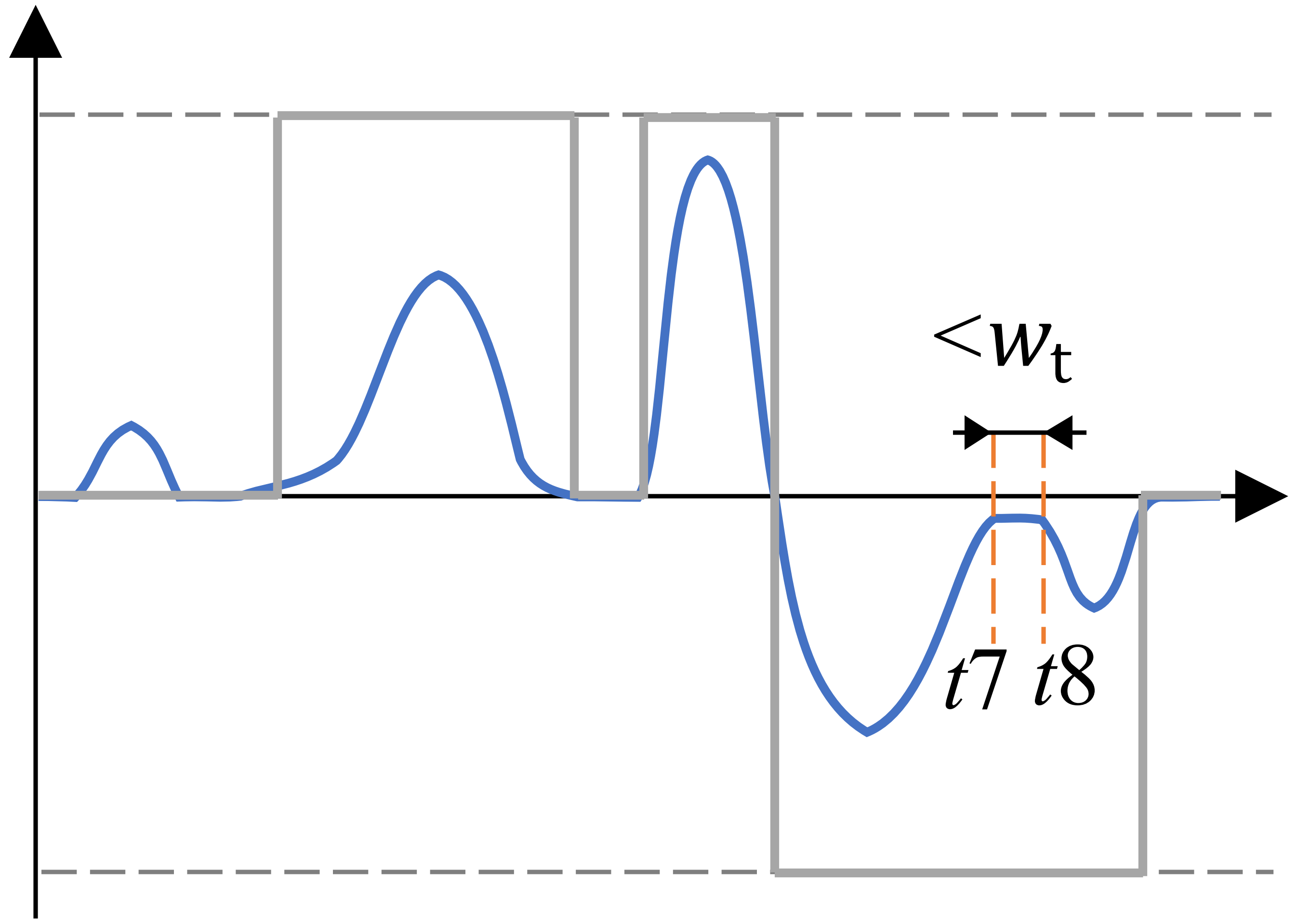

(e) Replacing shot-time motions.
