# Supplementary material for: Mitigating the Impact of Electrode Shift on Classification Performance in Electromyography Applications Using Sliding-Window Normalization
Source: Sensors (Basel). 2025 Jul 1;25(13):4119. doi: 10.3390/s25134119 (PMC12251759; doi:10.3390/s25134119)
Supplement: Supplementary file 1 [file sensors-25-04119-s001.zip › supplementary materials/FiguresResults/A_diffacc_swn vs dnn methods_scatter_outline.pdf]

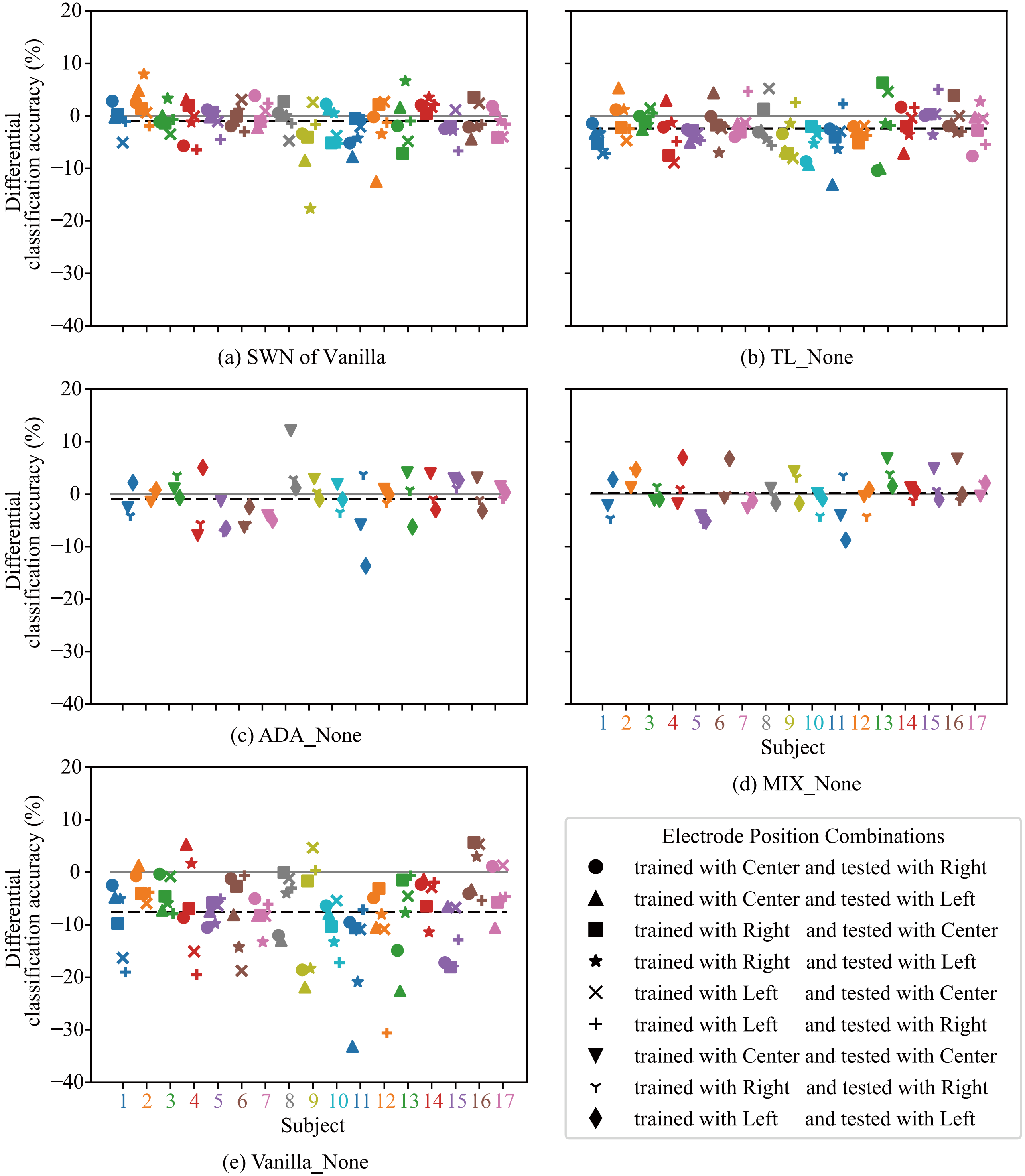

Differential classification accuracy (%)
20
10
0
−10
−20
−30
−40
(a) SWN of Vanilla
(b) TL_None
(c) ADA_None
1 2 3 4 5 6 7 8 9 10 11 12 13 14 15 16 17
Subject
(d) MIX_None
(e) Vanilla_None
Electrode Position Combinations
trained with Center and tested with Right
trained with Center and tested with Left
trained with Right and tested with Center
trained with Right and tested with Left
trained with Left and tested with Center
trained with Left and tested with Right
trained with Center and tested with Center
trained with Right and tested with Right
trained with Left and tested with Left
