# Supplementary material for: Mitigating the Impact of Electrode Shift on Classification Performance in Electromyography Applications Using Sliding-Window Normalization
Source: Sensors (Basel). 2025 Jul 1;25(13):4119. doi: 10.3390/s25134119 (PMC12251759; doi:10.3390/s25134119)
Supplement: Supplementary file 1 [file sensors-25-04119-s001.zip › supplementary materials/FiguresResults/B_diffacc_vs dnn methods with swn_scatter_outline.pdf]

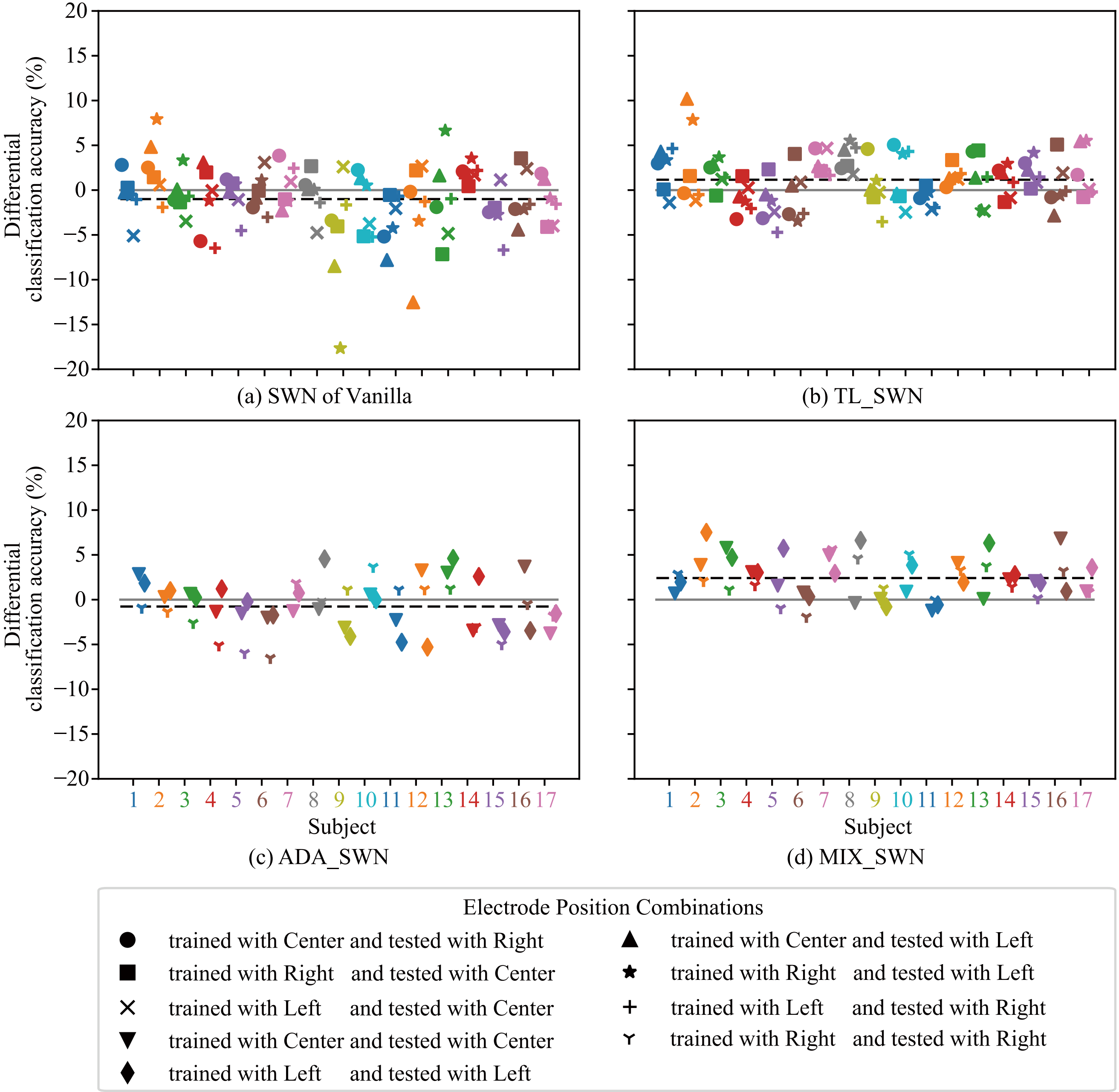

Differential classification accuracy (%)
20
15
10
5
0
−5
−10
−15
−20
(a) SWN of Vanilla
(b) TL_SWN
(c) ADA_SWN
(d) MIX_SWN
1 2 3 4 5 6 7 8 9 10 11 12 13 14 15 16 17
Subject
Electrode Position Combinations
trained with Center and tested with Right
trained with Right and tested with Center
trained with Left and tested with Center
trained with Center and tested with Center
trained with Left and tested with Left
trained with Center and tested with Left
trained with Right and tested with Left
trained with Left and tested with Right
trained with Right and tested with Right
