# Supplementary material for: Mitigating the Impact of Electrode Shift on Classification Performance in Electromyography Applications Using Sliding-Window Normalization
Source: Sensors (Basel). 2025 Jul 1;25(13):4119. doi: 10.3390/s25134119 (PMC12251759; doi:10.3390/s25134119)
Supplement: Supplementary file 1 [file sensors-25-04119-s001.zip › supplementary materials/supplementary materials.pdf]

S1 Comparison of Alternative Methods Against the SWN

In the Section 3.2, we compared the performance between SWN of Vanilla and alternative methods without normalization. In this section, we investigate the difference classification accuracy each subject between SWN of Vanilla and TL_None, ADA_None, MIX_None, and Vanilla_None (Figure S1). We compared the best performance results each method in the window lengths for normalization and feature extraction, like Section 3.2. The window lengths for normalization and feature extraction are changed in the range of 200-1000 ms in 200 ms increments. In the Figure S1, the black solid line shows mean and the gray line shows 0%.

From Figure S1, there are same performance behavior among subjects in the a-e. The difference class accuracy was best in the order of MIX_None, SWN of Vanilla, like in Section 3.2.

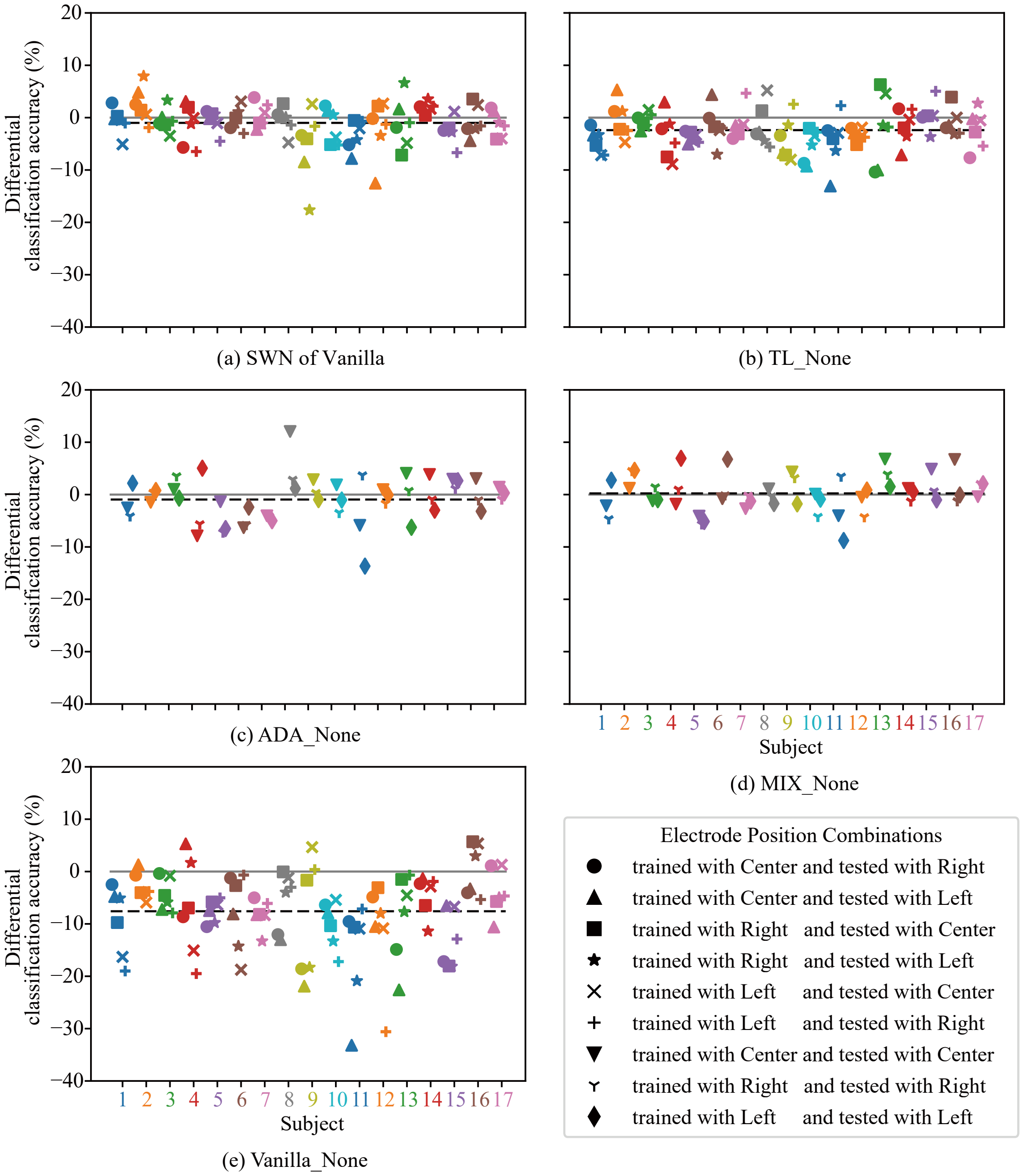

Figure S1: Performance comparison each subject between SWN and alternative methods. (a)-(e) indicate method name. The black solid lines indicate mean differential classification accuracy among electrode position combinations and subjects each method, and the gray line indicates 0%.

S2 Comparison of DNN methods with the SWN Integration

In the Section 3.3, we compared the performance among DNN methods with SWN. In this section, we investigate the difference classification accuracy each subject among DNN methods with SWN (Figure S2). In Figure S2, the black solid line shows mean and the gray line shows 0%.

From Figure S2, there are same performance behavior among subjects in the a-d. The difference class accuracy was best in the order of MIX_SWN, TL_SWN, like in Section 3.3.

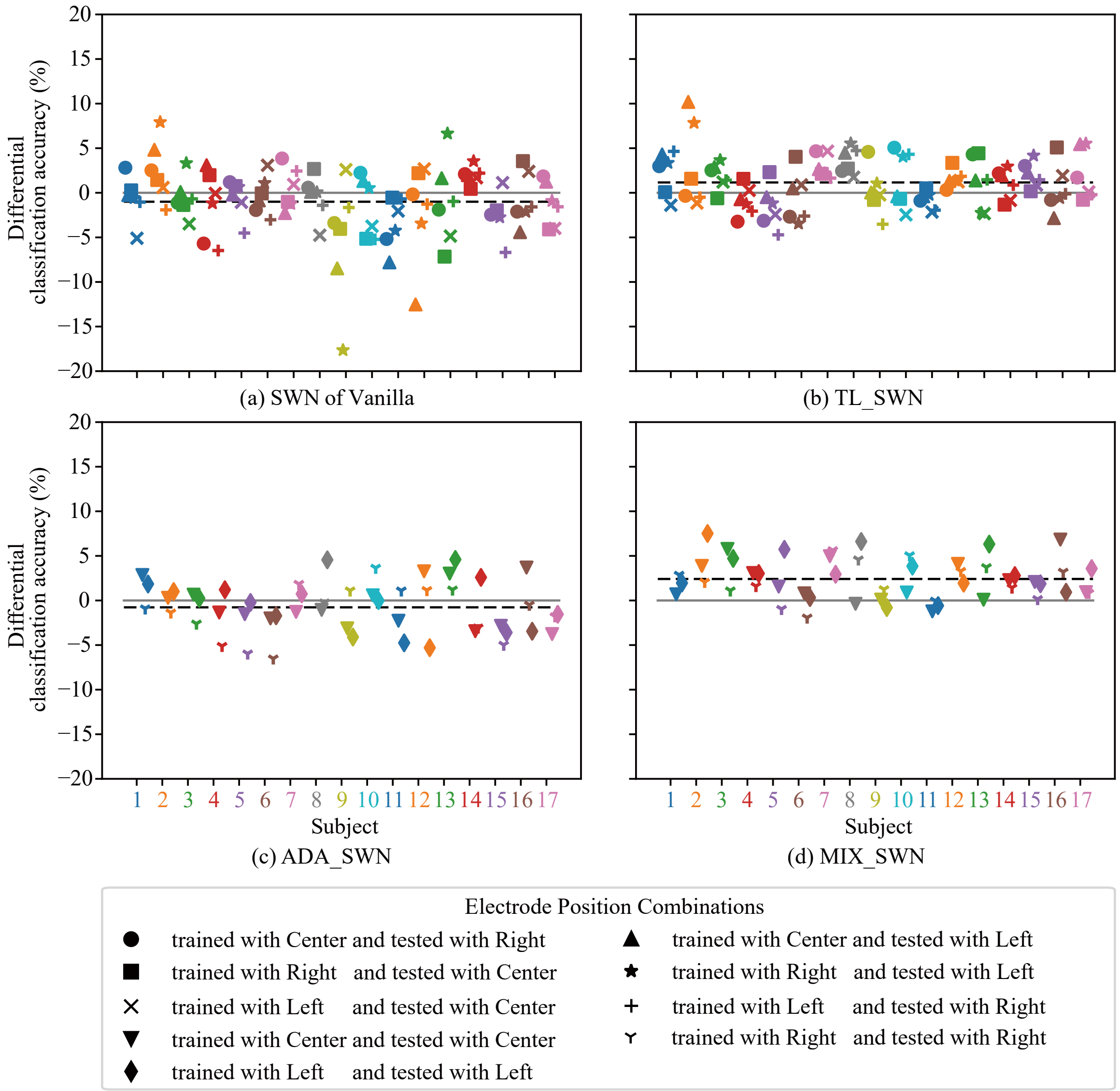

Figure S2: Performance comparison each subject between DNN methods with SWN and without normalization. (a)-(d) indicate DNN method name. The black solid lines indicate mean differential classification accuracy among electrode position combinations and subjects each method, and the gray line indicates 0%.

S3 Sensorless Motion Capture System

We use DeepLabCut-Live [1] and anipose [2] to capture human body part positions. We describe the how to get the elbow and shoulder joint angles from images, initializing multi-view triangulation, and initializing the rotating matrix and shifting vector.

S3.1 Discription of Sensorless Motion Capture System

We show the progress in getting the elbow and shoulder joint angles from images.

1. Calibrating for 6) Multi-view triangulation and 7) Coordinate transformation with a 5×5 checkerboard.

2. Capturing three images (20 Hz) by OpenCV (C++) with a video capture board.

3. Resizing images 1920×1080 to 640×360.

4. Sending resized images from C++ to Python.

5. Estimating body part positions in each image by DeepLabCut-Live.

6. Multi-view triangulation by anipose.

7. Coordinate transformation by Equation (S1) from the 3D triangulation space to the 3D human space. In the 3D triangulation space:D^{T}, the three-dimensional coordinate system is formed based on the camera's orientation and is therefore not necessarily horizontal with respect to the ground. To address this, we rotate the coordinate system and transform it into a three-dimensional coordinate system that is aligned with the ground: D^{H}, making it more convenient for human interpretation.

$$\boldsymbol{p}^{\mathrm{H}} \;=\; \mathrm{R}^{\mathrm{xyz}}\,\boldsymbol{p}^{\mathrm{T}} \;+\; \boldsymbol{t} \tag{S1}$$

8. Sending body part positions from Python to C++.

9. Transforming wrist, elbow, and shoulder positions to elbow and shoulder joint angles by Equation (S12).

S3.2 Initializing Multi-View Triangulation

In order to perform triangulation, the transformation parameters must be initialized. We show procedure that initializes multi-view triangulation (anipose).

1. Saving three synchronized videos showing a checkerboard. The checkerboard must be photographed at several angles and positions. This checkerboard was made by `cv2.aruco.CharucoBoard` class in OpenCV (Python), 5×5 grids, 0.17 m square length, 0.15 m marker length, 4 marker bits, and 50 marker kinds.

2. Initializing by anipose using saved videos.

S3.3 Initializing the Rotating Matrix and Shifting Vector

We describe the method for obtaining the rotation matrix: $\mathrm{R}^{\mathrm{xyz}}$ and translation vector: $\boldsymbol{t}$ used to transform the 3D triangulation space:D^{T} into a coordinate system that is horizontally aligned with the ground: D^{H}. Figure S3 indicates how to get the $\mathrm{R}^{\mathrm{xyz}}$ and $\boldsymbol{t}$. They are got by allow points ($\boldsymbol{x}^{\mathrm{C1\text{~}CN}}$, $\boldsymbol{y}^{\mathrm{C1\text{~}CN}}$, $\boldsymbol{z}^{\mathrm{C1\text{~}CN}}$) and origins ($\mathbf{O}^{\mathrm{C1\text{~}CN}}$) shown in the images. Here, N is the number of the cameras.

1. Getting the origin position of the checkerboard in the image for each camera. We used `detectBoard` function in the `cv2.aruco.CharucoDetector` class in the OpenCV in Python.

2. Getting ***Rvec*** and ***tvec*** to transform between D^{I} (2D image space) and D^{C} (3D camera space) for the origin of the checkerboard for each camera. We used `cv2.aruco.estimatePoseCharucoBoard` function in the OpenCV in Python. The ***Rvec*** is a vector to rotate the object coordinate to the camera coordinate, and the ***tvec*** is a vector to translate the origin of the object coordinate to the camera coordinate.

3. Projecting the arrow points: $\boldsymbol{x}^{\mathrm{C1\tilde{~}CN}}$, $\boldsymbol{y}^{\mathrm{C1\tilde{~}CN}}$, and $\boldsymbol{z}^{\mathrm{C1\tilde{~}CN}}$, and the origin of the checkerboard: $\mathbf{O}^{\mathrm{C1\tilde{~}CN}}$, form $\mathrm{D}^{\mathrm{C1\tilde{~}CN}}$ to $\mathrm{D}^{\mathrm{I1\tilde{~}IN}}$ by `cv2.projectPoints` function in OpenCV in Python. We set $\boldsymbol{x}^{\mathrm{C1\tilde{~}CN}}$, $\boldsymbol{y}^{\mathrm{C1\tilde{~}CN}}$, and $\boldsymbol{z}^{\mathrm{C1\tilde{~}CN}}$ as ...

$$\left[\boldsymbol{x}^{\mathrm{C1\tilde{~}CN}},\ \boldsymbol{y}^{\mathrm{C1\tilde{~}CN}},\ \boldsymbol{z}^{\mathrm{C1\tilde{~}CN}},\ \mathbf{O}^{\mathrm{C1\tilde{~}CN}}\right] = L^{\mathrm{a}} \begin{pmatrix} 0 & 1 & 0 & 0 \\ 1 & 0 & 0 & 0 \\ 0 & 0 & -1 & 0 \end{pmatrix} \tag{S2}$$

where, L^{a} is the arrow length of the checkerboard that was set to 0.2 [m]. We use the cmtx and dist that were acquired by anipose to conduct `cv2.projectPoints` function. Here, the cmtx means camera matrix which is a 3×3 matrix representing the intrinsic camera parameters and the dist means the parameters for correcting image distortion caused by camera lenses.

4. Multi-view triangulation by anipose to transform allow points: $\boldsymbol{x}^{\mathrm{T}}$, $\boldsymbol{y}^{\mathrm{T}}$, and $\boldsymbol{z}^{\mathrm{T}}$ and origin point of the checkerboard: $\boldsymbol{r}^{\mathrm{T}}$.

5. Getting the arrow vectors and a shifting vector.

$$\left[\bar{\boldsymbol{x}}^{\mathrm{T}},\ \bar{\boldsymbol{y}}^{\mathrm{T}},\ \bar{\boldsymbol{z}}^{\mathrm{T}}\right] = \left[\boldsymbol{x}^{\mathrm{T}},\ \boldsymbol{y}^{\mathrm{T}},\ \boldsymbol{z}^{\mathrm{T}}\right] - \boldsymbol{r}^{\mathrm{T}} \tag{S3}$$

Where, $\boldsymbol{x}^{\mathrm{T}}$, $\boldsymbol{y}^{\mathrm{T}}$, and $\boldsymbol{z}^{\mathrm{T}}$ indicates the arrow vectors. Further, the shifting vector is shown Equation (S4).

$$\boldsymbol{t} = -\boldsymbol{r}^{\mathrm{T}} \tag{S4}$$

6. Gram-Schmidt Cartesian Coordinate Transformation for arrow vectors. Transforming in the order $\bar{\boldsymbol{x}}^{\mathrm{T}} - \bar{\boldsymbol{y}}^{\mathrm{T}}$, $\bar{\boldsymbol{x}}^{\mathrm{T}} - \bar{\boldsymbol{z}}^{\mathrm{T}}$, $\bar{\boldsymbol{y}}^{\mathrm{T}} - \bar{\boldsymbol{z}}^{\mathrm{T}}$.

7. Getting coordinate rotation matrix. We find a coordinate rotation matrix, $\mathrm{R}^{\mathrm{xyz}}$, that satisfies Equation (S5).

$$\begin{gathered} \left[\bar{\boldsymbol{x}}^{\mathrm{H}} \quad \bar{\boldsymbol{y}}^{\mathrm{H}} \quad \bar{\boldsymbol{z}}^{\mathrm{H}}\right] = \mathrm{R}^{\mathrm{xyz}} \left[\bar{\boldsymbol{x}}^{\mathrm{T}} \quad \bar{\boldsymbol{y}}^{\mathrm{T}} \quad \bar{\boldsymbol{z}}^{\mathrm{T}}\right] \\ \bar{\boldsymbol{x}}^{\mathrm{H}} = \begin{bmatrix} 1 & 0 & 0 \end{bmatrix}^{\mathrm{T}} \\ \bar{\boldsymbol{y}}^{\mathrm{H}} = \begin{bmatrix} 0 & 1 & 0 \end{bmatrix}^{\mathrm{T}}, \quad \bar{\boldsymbol{z}}^{\mathrm{H}} = \begin{bmatrix} 0 & 0 & 1 \end{bmatrix}^{\mathrm{T}} \end{gathered} \tag{S5}$$

where, $\bar{\boldsymbol{x}}^{\mathrm{H}}$, $\bar{\boldsymbol{y}}^{\mathrm{H}}$, $\bar{\boldsymbol{z}}^{\mathrm{H}}$ are normalized $\boldsymbol{x}^{\mathrm{H}}$, $\boldsymbol{y}^{\mathrm{H}}$, $\boldsymbol{z}^{\mathrm{H}}$.

We rotate the vectors by the roll-pitch-yaw method as Equation (S6).

$$\begin{gathered} \mathrm{Rot}(\theta_{\mathrm{x}}, \theta_{\mathrm{y}}, \theta_{\mathrm{z}}) = \mathrm{Yaw}(\theta_{\mathrm{z}})\ \mathrm{Pitch}(\theta_{\mathrm{y}})\ \mathrm{Roll}(\theta_{\mathrm{x}}) \\ \mathrm{Roll}(\theta_{\mathrm{x}}) = \begin{pmatrix} 1 & 0 & 0 \\ 0 & \cos\theta_{\mathrm{x}} & -\sin\theta_{\mathrm{x}} \\ 0 & \sin\theta_{\mathrm{x}} & \cos\theta_{\mathrm{x}} \end{pmatrix} \\ \mathrm{Pitch}(\theta_{\mathrm{y}}) = \begin{pmatrix} \cos\theta_{\mathrm{y}} & 0 & \sin\theta_{\mathrm{y}} \\ 0 & 1 & 0 \\ -\sin\theta_{\mathrm{y}} & 0 & \cos\theta_{\mathrm{y}} \end{pmatrix} \\ \mathrm{Yaw}(\theta_{\mathrm{z}}) = \begin{pmatrix} \cos\theta_{\mathrm{z}} & -\sin\theta_{\mathrm{z}} & 0 \\ \sin\theta_{\mathrm{z}} & \cos\theta_{\mathrm{z}} & 0 \\ 0 & 0 & 1 \end{pmatrix} \end{gathered} \tag{S6}$$

The coordinate transforming is conducted by Equation (S7).

$$\mathrm{R}^{\mathrm{xyz}} = \mathrm{R}^{\mathrm{z}'}\,\mathrm{R}^{\mathrm{y}}\,\mathrm{R}^{\mathrm{x}} \tag{S7}$$

R^{x} is gotten from Equation (S8), R^{y} is gotten from Equation (S9), and $\mathrm{R}^{\mathrm{z}'}$ is gotten from Equation (S10).

Rotating x axis.

$$
\begin{aligned}
&\mathrm{R^x} = \mathrm{Rot}(0, \theta^1_\mathrm{y}, \theta^1_\mathrm{z})\\
&\theta^1_\mathrm{y} = \mathrm{atan2d}\Big(\mathrm{P}^1_\mathrm{xz},\, \mathrm{P}^1_\mathrm{xx}\Big)\\
&\theta^1_\mathrm{z} = \begin{cases} \mathrm{atan2d}\Big(\mathrm{P}^1_\mathrm{xy},\, \dfrac{\mathrm{P}^1_\mathrm{xx}}{\cos\theta^1_\mathrm{y}}\Big) \\ \qquad (-45^\circ < \theta^1_\mathrm{y} < 45^\circ \text{ or} \\ \qquad 135^\circ < \theta^1_\mathrm{y} < 225^\circ) \\ \mathrm{atan2d}\Big(\mathrm{P}^1_\mathrm{xy},\, -\dfrac{\mathrm{P}^1_\mathrm{xz}}{\sin\theta^1_\mathrm{y}}\Big) \quad (\text{otherwise}) \end{cases}\\
&\mathrm{P}^1 = \big[\bar{x}^\mathrm{T},\, \bar{y}^\mathrm{T},\, \bar{z}^\mathrm{T}\big]
\end{aligned} \tag{S8}
$$

Rotating y axis.

$$
\begin{aligned}
&\mathrm{R}^y = \mathrm{Rot}(\theta^2_\mathrm{x},\, 0,\, \theta^2_\mathrm{z})\\
&\theta^2_\mathrm{x} = \mathrm{atan2d}\Big(\mathrm{P}^2_\mathrm{yz},\, \mathrm{P}^2_\mathrm{yy}\Big)\\
&\theta^2_\mathrm{z} = \begin{cases} \mathrm{atan2d}\Big(\mathrm{P}^2_\mathrm{yx},\, \dfrac{\mathrm{P}^2_\mathrm{yy}}{\cos\theta^2_\mathrm{x}}\Big) \\ \qquad (-45^\circ < \theta^2_\mathrm{x} < 45^\circ \text{ or} \\ \qquad 135^\circ < \theta^2_\mathrm{x} < 225^\circ) \\ \mathrm{atan2d}\Big(\mathrm{P}^2_\mathrm{zz},\, -\dfrac{\mathrm{P}^2_\mathrm{yz}}{\sin\theta^2_\mathrm{x}}\Big) \quad (\text{otherwise}) \end{cases}\\
&\mathrm{P}^2 = \mathrm{R^x}\, \mathrm{P}^1
\end{aligned} \tag{S9}
$$

Rotating z axis.

$$
\begin{aligned}
&\mathrm{R}^{z\prime} = \begin{pmatrix} 1 & 0 & 0 \\ 0 & 1 & 0 \\ 0 & 0 & \mathrm{R^z_{zz}} \end{pmatrix}\\
&\mathrm{R^z} = \mathrm{Rot}(\theta^3_\mathrm{x},\, \theta^3_\mathrm{y},\, 0)\\
&\theta^3_\mathrm{x} = \mathrm{atan2d}\Big(\mathrm{P}^3_\mathrm{zx},\, \mathrm{P}^3_\mathrm{zz}\Big)\\
&\theta^3_\mathrm{y} = \begin{cases} \mathrm{atan2d}\Big(\mathrm{P}^3_\mathrm{zx},\, \dfrac{\mathrm{P}^3_\mathrm{zz}}{\cos\theta^2_\mathrm{x}}\Big) \\ \qquad (-45^\circ < \theta^3_\mathrm{x} < 45^\circ \text{ or} \\ \qquad 135^\circ < \theta^3_\mathrm{x} < 225^\circ) \\ \mathrm{atan2d}\Big(\mathrm{P}^3_\mathrm{zz},\, -\dfrac{\mathrm{P}^3_\mathrm{zy}}{\sin\theta^3_\mathrm{x}}\Big) \quad (\text{otherwise}) \end{cases}\\
&\mathrm{P}^3 = \mathrm{R^y}\, \mathrm{P}^2
\end{aligned} \tag{S10}
$$

S4 Generated Tasks

Subjects conducted 5 kinds of tasks that took approximately 60 s to run, included rest and task parts with 1:1.(Figure S4). These movements were made by the minimum jerk mode [3] with the border conditions. During the experiment, the length of the forearm and upper arm were varied because there is an accuracy

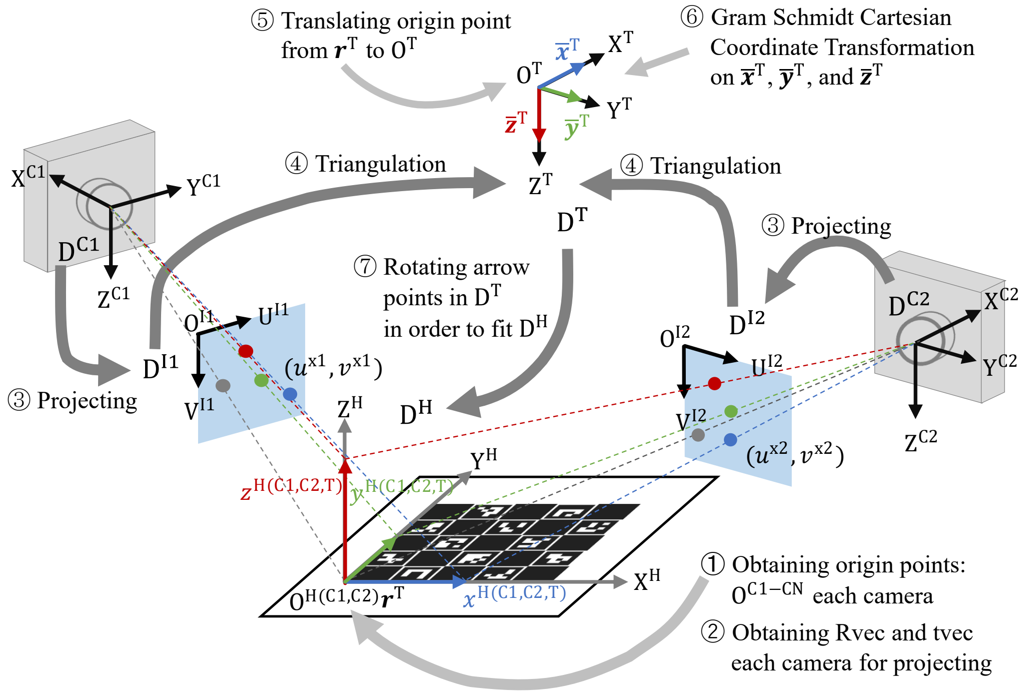

Figure S3: An explanation of the method for transforming arrow points from the 3D camera space into the 3D human space. D^H means a 3D space of the human, D^C means a 3D space of the camera, D^I means a 2D space of the image, and D^T means a 3D space after triangulation. the circled numbers regard to numbers in **Initializing the rotating matrix and shifting vector**. To obtain rotation matrix and translation vector to transform any points in D^T ioto D^H, the blue, green, and red arrow points and a origin point in D^C are obtained, and transformed into in order D^I, D^T. The translation vector is obtained by translating a origin point from O^T to O^H. Further, the rotation matrix is obtained by rotating three arrow points individually.

error in the sensorless motion capture system. Therefore, we calculate the target marker positions from prepared target angles and the current arm lengths as Equation (S11).

$$\begin{cases} x_t^{\mathrm{tgt}} = L_t^{\mathrm{sld}} \cos\left(\theta_t^{\mathrm{sld}}\right) + L_t^{\mathrm{elb}} \cos\left(\theta_t^{\mathrm{sld}} + \theta_t^{\mathrm{elb}}\right), \\ y_t^{\mathrm{tgt}} = L_t^{\mathrm{sld}} \sin\left(\theta_t^{\mathrm{sld}}\right) + L_t^{\mathrm{elb}} \sin\left(\theta_t^{\mathrm{sld}} + \theta_t^{\mathrm{elb}}\right) \end{cases} \tag{S11}$$

Where, x_t^{tgt} and y_t^{tgt} are wrist positions at the t time, L_t^{sld} and L_t^{sld} are the length of the forearm and upper arm at the t time, and θ_t^{sld} and θ_t^{elb} are the prepared target shoulder and elbow joint angles at the t time. We set the based target points hand-made and extended them by minimum jerk model (frame rate: 120 Hz). We got target joint angles assuming the length of the forearm and upper arm are 30 cm with Equation (S12).

$$\begin{cases} \theta^{\mathrm{sld}} = \mathrm{atan2d}(a,\, b) - \mathrm{atan2d}\left(\sqrt{a^2 + b^2 - c^2},\, c\right) \\ \theta^{\mathrm{elb}} = \mathrm{atan2d}\left(\sqrt{a^2 + b^2 - c^2},\, c\right) \\ \quad + \mathrm{atan2d}\left(\sqrt{a^2 + b^2 - d^2},\, d\right) \end{cases}$$
$$a = y^{wst} - y^{sld} \tag{S12}$$
$$b = x^{wst} - x^{sld}$$
$$c = \frac{a^2 + b^2 + L^{\mathrm{sld}\,2} - L^{\mathrm{elb}\,2}}{2\,L^{\mathrm{sld}}}$$
$$d = \frac{a^2 + b^2 - L^{\mathrm{sld}\,2} + L^{\mathrm{elb}\,2}}{2\,L^{\mathrm{elb}}}$$

We describe how to generate the 5 kinds of the tasks in Figure S4. These tasks include different motions, which are illustrated as distinct colored trajectories in Figure S4. For all segments of the motions, the start and end boundary conditions are defined such that the velocity and acceleration of the x and y axes are 0 rad./s and 0 rad./s^2, respectively. In particular, for the straight trajectories shown in Figure S4A, C, and D, the boundary conditions are also set so that the velocity and acceleration in the x and y axes are 0 rad./s and 0 rad./s^2. Further, in Figure S4E, the curved trajectories are got based on the elbow or shoulder joint angle rather than the wrist position.

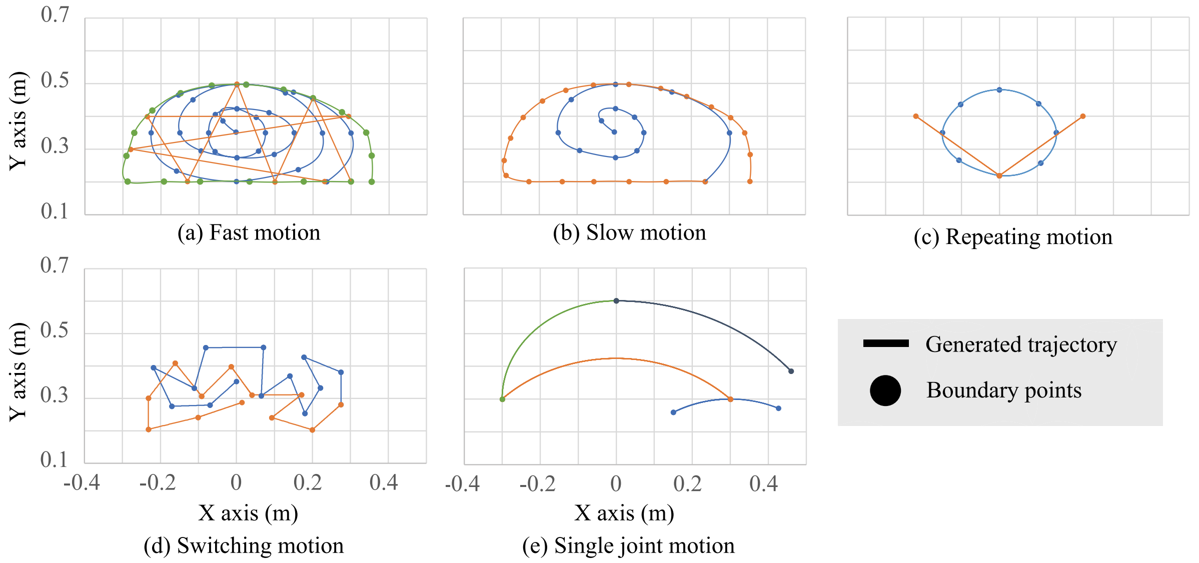

Figure S4: The 5 kinds of the designed tasks. The generated trajectory is obtained by minimum jerk model with boundary conditions: x and y positions, velocity, acceleration, and time. Further, These tasks include different motions, which are illustrated as distinct colored trajectories.

S5 Motion Labels Processing

We describe how to get motion labels from elbow joint angular velocity. First, we get the elbow joint angular velocity with Equation (S12) and he difference method. The motion labels are got by 5 steps (Figure S5). 1st step is motion labeling based on the elbow joint angular velocity by Equation (S13).

$$l_t = \begin{cases} \text{flexion} & (\omega_t > th_{\omega 1}) \\ \text{extension} & (\omega_t < -th_{\omega 1}) \\ \text{rest} & (\text{otherwise}) \end{cases} \tag{S13}$$

Here, l_t is a motion label at t time, ω_t is an elbow joint angular velocity at t time, $th_{\omega 1}$ is a threshold for elbow joint angular velocity at t time. We set $th_{\omega 1}$ is 3.0 rad./s.

2nd step replaces the movement labels to rest if the time width of the movement is under $w_t$ms by Equation (S14).

$$l_{t1\sim t2} = \text{rest} \quad (t2 - t1 > w_t) \tag{S14}$$

Where, $t1$ is the start time for flexion or extension, $t2$ is the end time for flexion or extension. $t1$ and $t2$ are defined by the elbow joint angular velocity is over $th_{\omega 1}$. We set w_t is 200ms.

3rd step extends the movement time width on both ends between $t1$ and $t2$ in order to detect the onset of a movement at an early stage and predict the moment when it fully concludes by Equation (S15).

$$\begin{aligned} l_{t3\sim t1} &= l_{t1} \\ l_{t2\sim t4} &= l_{t2} \end{aligned} \tag{S15}$$

Where, $t3$ is a time that is $|\omega_t| > th_\omega$ and close to $t1$. $t4$ is a time that is $|\omega_t| > th_\omega$ and close to $t2$.

4th step change the rest to continuous movement in order to prevent the instant at which the type of movement changes from being misclassified as rest by Equation (S16).

$$l_{t5\sim t6} = \begin{cases} \text{flexion} & (\omega_t \geq th_{\omega 2} \text{ and } s \geq \mathrm{th_s}) \\ \text{extension} & (\omega_t < -th_{\omega 2} \text{ and } s \geq \mathrm{th_s}) \\ \text{unchange} & (\text{otherwise}) \end{cases} \tag{S16}$$

Where ω_t is an elbow joint angular velocity at t time, s is a sloop between $t5$ and $t6$, $\mathrm{th_s}$ is a threshold for the sloop, $t5$ and $t6$ are start and end time for rest. We set $th_{\omega 2}$ is 1.0 rad./s and $\mathrm{th_s}$ is 5 rad./s^2 and got s by minimum square method.

The final step replaces the motion labels to the former or post motion label if the time width of the motion label is under w_t ms in order to exclude motions that are shorter than the predefined duration by Equation (S17).

$$l_{t7\sim t8} = \begin{cases} l_{t7-1} & (t7 - 1 \geq 0) \\ l_{t8+1} & (\text{otherwise}) \end{cases} \tag{S17}$$

Where $t7$ and $t8$ are both ends between one of the motions.

References

[1] Kane, G.; Lopes, G.; Sanders, J.; Mathis, A.; Mathis, M. Real-time, low-latency closed-loop feedback using markerless posture tracking. *eLife* **2020**. Available online: `https://github.com/DeepLabCut/DeepLabCut-live` (accessed on 30 May 2025).

[2] Karashchuk, P.; Rupp, K.L.; Dickinson, E.S.; Walling-Bell, S.; Sanders, E.; Azim, E.; Brunton, B.W.; Tuthill, J.C. Anipose: A toolkit for robust markerless 3d pose estimation. *Cell Rep.* **2021**, *36*. Available online: `https://anipose.readthedocs.io/en/latest/` (accessed on 30 May 2025).

[3] Flash, T.; Hogan, N. The coordination of arm movements: an experimentally confirmed mathematical model. *J. Neurosci.* **1985**, *5*, 1688–1703, .

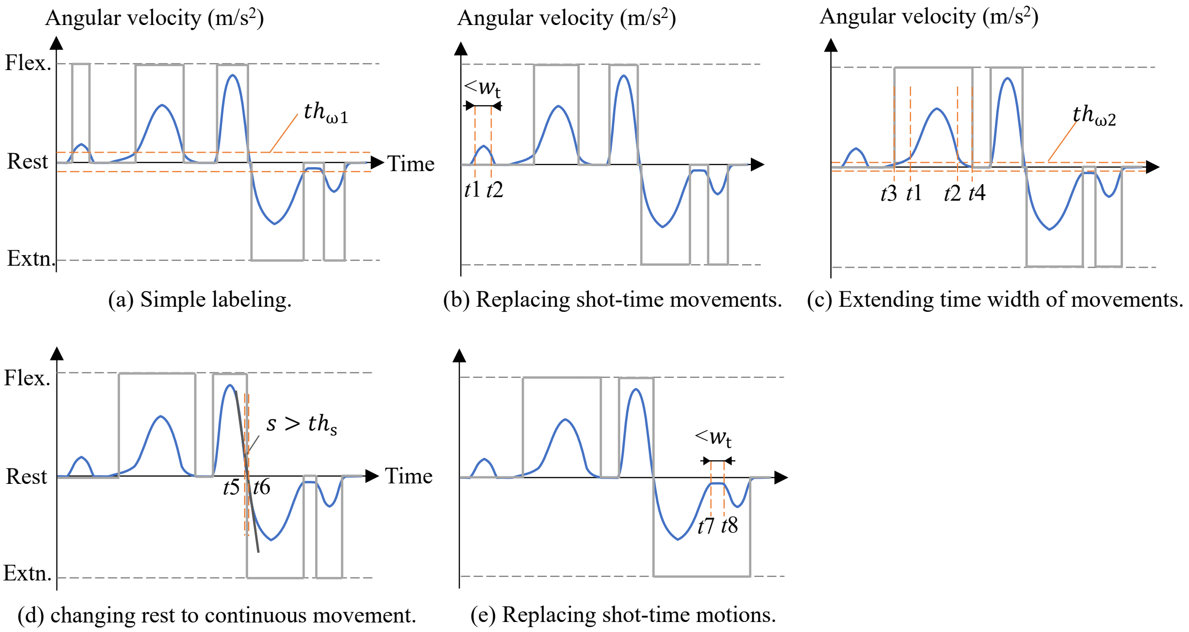

(a) Simple labeling. (b) Replacing shot-time movements. (c) Extending time width of movements.

(d) changing rest to continuous movement. (e) Replacing shot-time motions.

Figure S5: The motion labels processing. (a)-(e) indicate processing names. the gray lines mean motion labels. The blue lines mean angular velocity.
