# Supplementary figures and images for: Mitigating the Impact of Electrode Shift on Classification Performance in Electromyography Applications Using Sliding-Window Normalization
Source: Sensors (Basel). 2025 Jul 1;25(13):4119. doi: 10.3390/s25134119 (PMC12251759; doi:10.3390/s25134119)

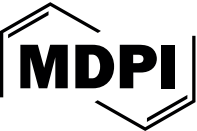

Supplement: Supplementary file 1 [file sensors-25-04119-s001.zip › supplementary materials/Definitions/logo-mdpi-eps-converted-to.pdf]

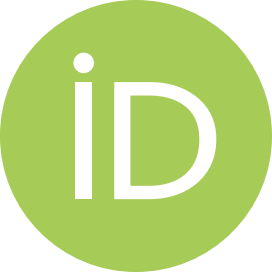

Supplement: Supplementary file 1 [file sensors-25-04119-s001.zip › supplementary materials/Definitions/logo-orcid.pdf]

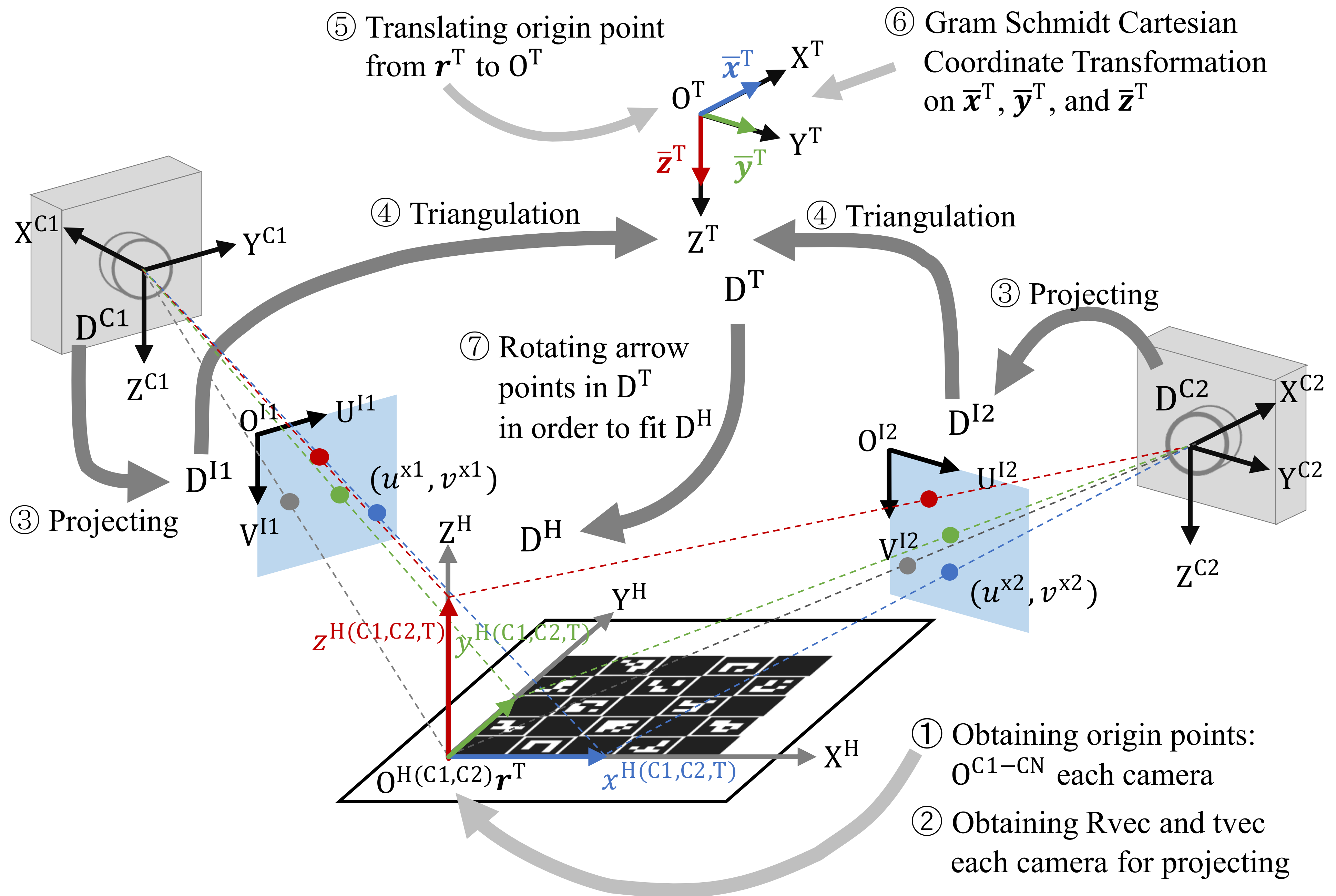

Supplement: Supplementary file 1 [file sensors-25-04119-s001.zip › supplementary materials/FiguresMethods/Sensorless Motion Capture System.pdf]

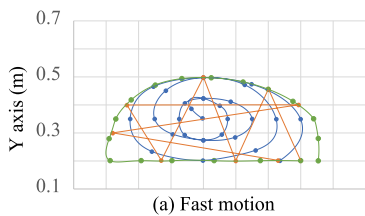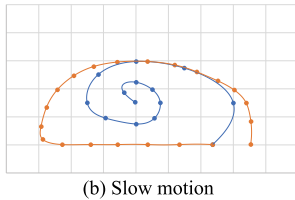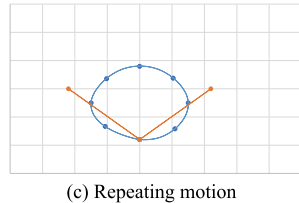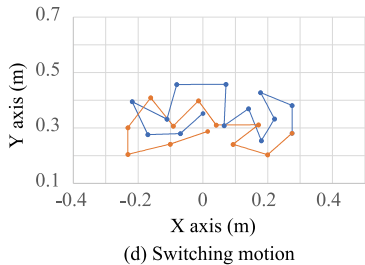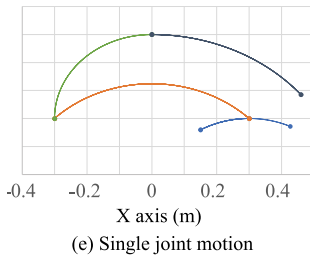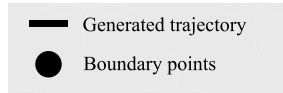

Supplement: Supplementary file 1 [file sensors-25-04119-s001.zip › supplementary materials/FiguresMethods/Task2.pdf]
